# Design of Promising Heptacoordinated Organotin (IV) Complexes-PEDOT: PSS-Based Composite for New-Generation Optoelectronic Devices Applications

**DOI:** 10.3390/polym13071023

**Published:** 2021-03-25

**Authors:** María Elena Sánchez-Vergara, Leon Hamui, Elizabeth Gómez, Guillermo M. Chans, José Miguel Galván-Hidalgo

**Affiliations:** 1 Facultad de Ingeniería, Universidad Anáhuac México, Avenida Universidad Anáhuac 46, Col. Lomas Anáhuac, Huixquilucan 52786, Estado de México, Mexico; elena.sanchez@anahuac.mx; 2 Instituto de Química, Universidad Nacional Autónoma de México, Circuito Exterior s/n. C.U., Alcaldia Coyoacán, Ciudad de México 04510, Mexico; guille_chans@outlook.com (G.M.C.); chimeco1983@gmail.com (J.M.G.-H.)

**Keywords:** PEDOT:PSS, organotin(IV) complexes, thin film, bandgap, devices, electrical properties

## Abstract

The synthesis of four mononuclear heptacoordinated organotin (IV) complexes of mixed ligands derived from tridentated Schiff bases and pyrazinecarboxylic acid is reported. This organotin (IV) complexes were prepared by using a multicomponent reaction, the reaction proceeds in moderate to good yields (64% to 82%). The complexes were characterized by UV-vis spectroscopy, IR spectroscopy, mass spectrometry, ^1^H, ^13^C, and ^119^Sn nuclear magnetic resonance (NMR) and elemental analysis. The spectroscopic analysis revealed that the tin atom is seven-coordinate in solution and that the carboxyl group acts as monodentate ligand. To determine the effect of the substituent on the optoelectronic properties of the organotin (IV) complexes, thin films were deposited, and the optical bandgap was obtained. A bandgap between 1.88 and 1.98 eV for the pellets and between 1.23 and 1.40 eV for the thin films was obtained. Later, different types of optoelectronic devices with architecture “contacts up/base down” were manufactured and analyzed to compare their electrical behavior. The design was intended to generate a composite based on the synthetized heptacoordinated organotin (IV) complexes embedded on the poly(3,4-ethylenedyoxithiophene)-poly(styrene sulfonate) (PEDOT:PSS). A Schottky curve at low voltages (<1.5 mV) and a current density variation of as much as ~3 × 10^−5^ A/cm^2^ at ~1.1 mV was observed. A generated photocurrent was of approximately 10^−7^ A and a photoconductivity between 4 × 10^−9^ and 7 × 10^−9^ S/cm for all the manufactured structures. The structural modifications on organotin (IV) complexes were focused on the electronic nature of the substituents and their ability to contribute to the electronic delocalization via the π system. The presence of the methyl group, a modest electron donor, or the non-substitution on the aromatic ring, has a reduced effect on the electronic properties of the molecule. However, a strong effect in the electronic properties of the material can be inferred from the presence of electron-withdrawing substituents like chlorine, able to reduce the gap energies.

## 1. Introduction

In recent years, the study of optoelectronic devices, based on organic semiconductors, such as organic photovoltaics (OPVs) and organic light-emitting diodes (OLEDs) has gained the interest of numerous researchers around the globe [1] due to their mechanical flexibility, lightweight, and prospective potential for roll-to-roll manufacturing and low cost [2,3]. The organic thin-film diode, first described in 1987, provides a simple example of a semiconductor device comprise by metal-semiconductor-metal (MSM) structure, containing a single layer of semiconducting polymer deposited in solution and sandwiched between metal contacts with different work functions [4,5]. In addition, there has been an upsurge in research regarding polymer electronics such as polymer light-emitting diodes (PLEDs) due to their lightweight, high flexibility, and solution-processable properties [6,7]. The materials required for the fabrication of these organic electronic devices (OEDs) in its most simple form, include a transparent substrate, a transparent electrode, a light-absorbing organic active layer, and a counter-electrode. The most common substrate used in the fabrication of OEDs is glass, due to its low-cost, commercial accessibility and its ability to protect the device against oxygen and water. The Indium–tin-oxide (ITO) is usually added on top of the substrate to act as the transparent electrode [3]. However, ITO is limited by its lack of mechanical flexibility and the lack of indium abundance on earth [2,8,9]. Because of this, various materials such as graphene and conducting polymers have been researched with the intent of replacing the ITO [2,10,11,12,13,14]. Poly(3,4-ethylenedyoxithiophene)-poly(styrene sulfonate), or PEDOT:PSS, has shown to be pretty successful as a replacement due to its high mechanical flexibility, excellent thermal stability [15,16], high transparency in the visible range and its ability to be dispersed in organic solvents such as water. Which in turn allows the fabrication of films by spin-coating or inkjet printing [2,17,18,19]. The PSS on the PEDOT:PSS allows it to be dispersed in solvents, but its conductivity is much lower than that of ITO by about three orders of magnitude, which makes it less appropriate for optoelectronic applications [2,6,17]. Therefore, the characteristics of PEDOT:PSS should be modified, by applying physical or chemical post-deposition treatments, to increase its conductivity [2,20,21,22]. Some ways in which these properties can be increased include solvent treatment [20,23,24,25], doping with additional organic compounds, with boiling points higher than that of the water, into the aqueous solution [2], and adding ionic surfactants into the aqueous solution [26].

It is also worth to mention that an important part of the OEDs architecture is the active layer, which often utilizes a fraction of the small semiconductor molecules (MSCs), because of the various electrical, optical, and stability requirements demanded [3]. Between the MSCs, metal-ion complexes have been studied by means of their molecular structures, magnetic, optical and electronic properties. Also, their applications in catalysis, supramolecular chemistry, biology and biochemistry. The metals can act as Lewis acids and form complexes with a variety of Lewis bases, where the metallic complex consist of a central metallic atom bonded to a ligand, in coordination chemistry. Furthermore, the type of ligand used as well as its denticity are of particular interest, due to the type of arrangements around the metal ion, coordination numbers and oxidation states that are possible to obtain for different applications. The nature of the donor atoms is also of important interest on the metal complexes structure, their properties, and potential applications [27,28]. The synthesis feasibility of Schiff base ligands makes them useful in materials chemistry. The polyfunctional groups can easily be added giving rise to a unique structural metallic array with an abundance of applications [29,30,31,32,33,34]. Among them, the organotin complexes, that have received much attention in view of their significant structural possibilities and a wide range of biological activities, such as antiviral, anticancer, antibacterial, antifungal, and anti-inflammatory; have been associated to the organic fragment nature bonded to the tin atom [35,36]. Regarding this, organotin (IV) carboxylates are fascinating because of the wide range of structures that they can be found, like monomers, dimers, tetramers, polymers, oligomers ladders, cyclic and hexameric drums. Their structural diversity has been related to the coordination modes and angles of the COO moiety [37,38]. Additionally, carboxylates are excellent building blocks in the formation of metal-coordination polymers, macrocycles and cages [39,40]. Organotin also possess a wide range of medicinal and industrial applications, as potential agents in cancer chemotherapy, catalysis of urethane, PVC stabilization, silicone curing and antifouling [41,42,43,44].

Despite the number of reports in the literature regarding the Schiff bases metal complexes exhibiting non-linear optical (NLO) properties [45,46], few investigations have been devoted to study their optical and electrical properties. Keeping this in mind in the present paper we describe the synthesis and the complete characterization of heptacoordinated organotin (IV) complexes from mixed ligands derived from tridentated Schiff bases ligands and pyrazinecarboxylic acid, obtained by a multicomponent reaction as synthetic strategy. Our research question is focused on knowing and analyzing what the effect of the peripheral substituent in the heptacoordinated organotin (IV) complexes base composite is, on the optoelectronic behavior. To answer the above, this work is divided into two parts: the first one corresponds to the synthesis and characterization of the heptacoordinated organotin (IV) complexes and the second one, refers to the manufacture of optoelectronic devices and their characterization. The design was intended to generate a composite based on the synthetized heptacoordinated organotin (IV) complexes embedded on the PEDOT:PSS. The effect of the substituent in the heptacoordinated organotin (IV) complexes when incorporated as active layer in the devices, was evaluated. The devices were manufactured using the high vacuum evaporation and spin-coating techniques, on transparent glass substrates. PEDOT:PSS polymer and zinc-hexadecafluoro-phthalocyanine (ZnPcF_16_) were included for hole carrier layers and electrons carrier layers, respectively. The ZnPcF_16_ is rich in π electrons and once the charge is at the molecule, it is quickly delocalized on the π system. The fluorine atoms induce a higher electron affinity to the entire molecule of ZnPcF_16_, which leads to a much more reactive system [47,48]. Interfacial films were included to favor contact between the organotin complexes and the electrodes, having a substantial impact on the extraction and collection processes of charges.

## 2. Materials and Methods

All reagents and solvents were obtained from commercial suppliers (Merck KGaA, Darmstadt, Germany) and used without further purification.

**Figure 1:**
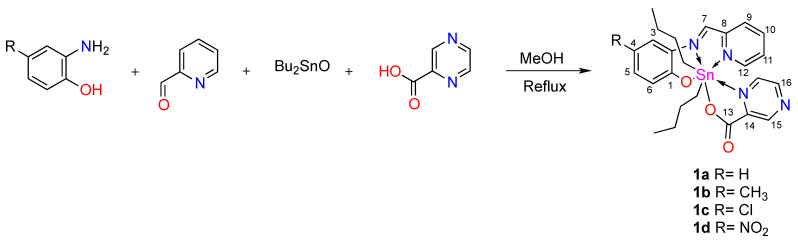
Synthesis of heptacoordinated organotin (IV) complexes.

Melting points of the complexes were measured with a Fischer-Johns MEL-TEMP II (Thermo Scientific, Waltham, MA, USA) apparatus and were not corrected. Conductivity was measured with a Hanna 6484 equipment (Hanna Instruments, Woonsocket, RI, USA) using anhydrous methanol as solvent. The UV-Vis absorption spectra were obtained with a Shimadzu UV-160U spectrometer (Shimadzu Scientific Instruments, Kyoto, Japan) in methanol solution (2.04530 × 10^−5^ M). The complexes infrared (IR) spectra were recorded with a Bruker Tensor 27 spectrometer (BRUKER, Ettlingen, Germany) using KBr. ^1^H, ^13^C and ^119^Sn NMR spectra were recorded with a Bruker Advance III spectrometer (BRUKER, Rheinstetten Germany) at 300.52, 75.57 and 118.8 MHz respectively, using chloroform-*d*. Chemical shifts (ppm) are relative to tetramethylsilane and the coupling constants are expressed in Hz. Two-dimensional COSY, HSQC and HMBC experiments were carried out to corroborate the assignment of ^1^H and ^13^C signals, according to the numeration depicted in Figure 1. The FAB+ (Fast Atom Bombardment) mass spectra were recorded with a JEOL-JMS-X103 spectrometer (JEOL, Tokyo, Japan) and the elemental analysis was conducted using a Vario micro instrument (Elementar, Langenselbold Hessen, Germany). During the deposition processes, the films’ thicknesses were monitored with a quartz crystal monitor. The FTIR analysis was performed on a Nicolet iS5-FT spectrometer (Thermo Fisher Scientific Inc., Waltham, MA, USA), for the films deposited on silicon wafers. For SEM, a ZEISS EVO LS 10 scanning electron microscope (Zeiss International Inc., Göttingen, Germany), operated at a voltage of 20 kV and a focal distance of 25 mm, was used for films deposited on Corning glass substrates. The optical absorption of the films on Corning glass substrates was measured on a Unicam spectrophotometer model UV300 (Thermo Fisher Scientific Inc., Waltham, MA, USA), in a 200–1100 nm wavelength range. The current-voltage-luminance characterization was performed using a programmable voltage source, a sensing station with lighting controller circuits from Next Robotix (Comercializadora K Mox, S.A. de C.V., CDMX, Mexico) and an auto-ranging Keithley 4200-SCS-PK1 pico-ammeter (Tektronix Inc., Beaverton, OR, USA).

### 2.1. General Procedure for Synthesis of Heptacoordinated Organotin (IV) Complexes 1a–1d

For the solution of the 2-pyridinecarboxaldehyde in 30 mL of methanol, the corresponding substituted *o*-aminophenol, dibutyltin oxide, and pyrazinecarboxylic acid were added in stochiometric ratio. The reaction mixture was stirred and refluxed for 8 h, then, the solvent was eliminated under reduced pressure to provide the desired complexes as amorphous solids. The products were purified by methanol crystallization. All complexes were soluble in organic solvents like methanol, ethanol, dimethyl sulfoxide (DMSO), chloroform and dichloromethane.

#### 2.1.1. 5,5-Di-n-butyl-1,4-diaza-6-oxatetracyclo [7.12.0.0^3,8^0^3,4^]-5-spirostanna-20,23-diaza-17-oxabyciclo-19,20,21-triene [0.0.0^19,20^] heptadeca-3,7,9,11,13,15-hexaene (1a)

Compound **1a** was prepared from 0.1126 g of 2-pyridinecarboxaldehyde (1.0512 mmol), 0.1147 g of 2-aminophenol (1.0512 mmol), 0.1304 g of pyrazinecarboxylic acid (1.0512 mmol), and 0.2617 g of dibutyltin oxide (0.8034 mmol). 0.4154 g (71%) orange solid; m.p. 218–220 °C (dec.); molar conductance, Λ_M_ (1 × 10^−3^ M, methanol): 28.6 ohm^−1^ cm^2^ mol^−1^ (non-electrolyte); UV-Vis [methanol, λ_max_/nm (ε/ M^−1^, cm^−1^)]: 219 (15206), 270 (10463) π-π* (aromatic), 335 (7676) π-π* (C=N), 459 (6845) n-π* (C=N); IR (KBr) cm^−1^: 1637 *ν_asym_*(COO), 1585 *ν*(C=N), 1351 *ν_sym_*(COO), 1141 *ν*(C-O_Arom_), 535 *ν*(Sn-C), 516 *ν*(Sn-O), 416 *ν*(Sn-N); ^1^H NMR (300.52 MHz, CDCl_3_)δ: 0.51 (6H, t, *J* = 7.21 Hz, H-δ), 0.88-1.00 (8H, m, H-β, γ), 1.09-1.18 (4H, m, H-α), 6.66 (1H, ddd, *J* = 1.50 Hz, *J* = 6.91 Hz, *J* = 6.91 Hz, H-4), 7.07 (1H, dd, *J* = 1.20 Hz, *J* = 8.41 Hz, H-6), 7.32 (1H, ddd, *J* = 1.50 Hz, *J* = 6.91 Hz, *J* = 6.91 Hz, H-5), 7.58 (1H, dd, *J* = 1.80 Hz, *J* = 8.41 Hz, H-3), 7.62 (1H, ddd, *J* = 1.20 Hz, *J* = 5.10 Hz, *J* = 5.10 Hz, H-11), 7.81 (1H, d, *J* = 7.81 Hz, H-9), 8.05 (1H, ddd, *J* = 1.50 Hz, *J* = 7.51 Hz, *J* = 7.51 Hz, H-10), 8.85 (1H, s, ^3^*J*(^1^H-^119/117^Sn) = 13.22 Hz, H-7), 8.99 (1H, d, *J* = 2.70 Hz, H-16), 9.50 (1H, dd, *J* = 1.20 Hz, *J* = 2.70 Hz, H-17), 9.74 (1H, d, *J* = 1.20 Hz, H-15), 9.77 (1H, d, *J* = 4.80 Hz, H-12); ^13^C NMR (75.57 MHz, CDCl_3_)δ: 166.3 (C-13), 164.7 (C-1), 150.9 (C-12), 148.2 (C-15), 148.0 (C-8), 147.5 (C-16), 144.3 (C-14), 142.3 (C-7), 141.7 (C-17), 139.4 (C-10), 133.7 (C-6), 130.0 (C-5), 126.5 (C-11), 126.4 (C-9), 121.8 (C-3), 116.3 (C-2), 115.5 (C-4), 32.4 (C-α, ^1^*J*(^119/117^Sn-^13^C) = 1101.15, 1054.96.15 Hz), 27.7 (C-γ, ^3^*J*(^119^Sn-^13^C) = 47.61 Hz), 26.1 (C-β, ^2^*J*(^119^Sn-^13^C) = 181.36 Hz, ^2^*J*(^117^Sn-^13^C) = 172.30 Hz), 13.9 (C-δ); ^119^Sn NMR (112.07 MHz, CDCl_3_)δ: −424; MS: (FAB^+^) [*m/z*] (%): 497 [M^+^- Bu] (21), 431 [M^+^- C_4_H_4_N_2_COO] (100), 317 [M^+^- C_4_H_4_N_2_COO - 2Bu] (45); Anal. Calcd. for C_25_H_30_N_4_O_3_Sn: C, 54.27; H, 5.47; N, 10.13. Found: C, 53.90; H, 5.55; N, 9.84.

#### 2.1.2. 5,5-Di-n-butyl-10-methyl-1,4-diaza-6-oxatetracyclo [7.12.0.0^3,8^0^3,4^]-5-spirostanna-20,23-diaza-17-oxabyciclo-19,20,21-triene [0.0.0^19,20^] heptadeca-3,7,9,11,13,15-hexaene (1b)

Compound **1b** was prepared from 0.1126 g of 2-pyridinecarboxaldehyde (1.0512 mmol), 0.1294 g of 2-amino-4-methylphenol (1.0512 mmol), 0.1304 g of pyrazinecarboxylic acid (1.0512 mmol), and 0.2617 g of dibutyltin oxide (0.8034 mmol). 0.4252 g (71%) dark red solid; m.p. 200–202 °C (dec.); molar conductance, Λ_M_ (1 × 10^−3^ M, methanol): 31.8 ohm^−1^ cm^2^ mol^−1^ (non-electrolyte); UV-Vis [methanol, λ_max_/nm (ε/ M^−1^, cm^−1^)]: 212 (17308), 269 (6510) π-π* (aromatic), 336 (10561) π-π* (C=N), 479 (8947) n-π* (C=N); IR (KBr) cm^−1^: 1639 *ν_asym_*(COO), 1584 *ν*(C=N), 1347 *ν_sym_*(COO), 1142 *ν*(C-O_Arom_), 552 *ν*(Sn-C), 511 *ν*(Sn-O), 415 *ν*(Sn-N); ^1^H NMR (300.52 MHz, CDCl_3_) δ: 0.51 (6H, t, *J* = 7.21 Hz, H-δ), 0.88-1.00 (8H, m, H-β, γ), 1.09–1.17 (4H, m, H-α), 2.14 (3H, s, H-18), 6.98 (1H, d, *J* = 8.41 Hz, H-6), 7.16 (1H, dd, *J* = 1.80 Hz, *J* = 8.41 Hz, H-5), 7.37 (1H, s, H-3), 7.60 (1H, ddd, *J* = 1.20 Hz, *J* = 5.10 Hz, *J* = 5.10 Hz, H-11), 7.78 (1H, d, *J* = 7.81 Hz, H-9), 8.05 (1H, dt, *J* = 1.50 Hz, *J* = 7.51 Hz, H-10), 8.80 (1H, s, ^3^*J*(^1^H-^119/117^Sn) = 13.50 Hz, H-7), 8.97 (1H, d, *J* = 2.70 Hz, H-16), 9.48 (1H, s_Broad_, H-17), 9.72 (1H, d, *J* = 1.20 Hz, H-15), 9.75 (1H, d, *J* = 4.80 Hz, H-12); ^13^C NMR (75.57 MHz, CDCl_3_)δ: 166.4 (C-13), 162.8 (C-1), 150.9 (C-12), 148.13 (C-15), 148.10 (C-8), 147.5 (C-16), 144.3 (C-14), 141.7 (C-17), 141.5 (C-7), 139.4 (C-10), 135.2 (C-5), 129.4 (C-2), 126.22 (C-9), 126.21 (C-11), 124.9 (C-4), 116.0 (C-3), 32.4 (C-α, ^1^*J*(^119/117^Sn-^13^C) = 1073.84, 767.03 Hz), 27.7 (C-γ, ^3^*J*(^119^Sn-^13^C) = 47.70 Hz), 26.1 (C-β, ^2^*J*(^119^Sn-^13^C) = 180.60 Hz), 20.7 (CH_3_), 13.9 (C-δ, ^4^*J*(^119^Sn-^13^C) = 13.60 Hz); ^119^Sn NMR (112.07 MHz, CDCl_3_)δ: -423; MS: (FAB^+^) [*m/z*] (%): 511 [M^+^ -Bu] (7), 445 [M^+^- C_4_H_4_N_2_COO] (31), 331 [M^+^- C_4_H_4_N_2_COO - 2Bu] (15); Anal. Calcd. for C_26_H_32_N_4_O_3_Sn: C, 55.05; H, 5.69; N, 9.88. Found: C, 54.75; H, 5.64; N, 9.75.

#### 2.1.3. 5,5-Di-n-butyl-10-chloro-1,4-diaza-6-oxatetracyclo [7.12.0.0^3,8^0^3,4^]-5-spirostanna-20,23-diaza-17-oxabyciclo-19,20,21-triene [0.0.0^19,20^] heptadeca-3,7,9,11,13,15-hexaene (1c)

Compound **1c** was prepared from 0.1126 g of 2-pyridinecarboxaldehyde (1.0512 mmol), 0.1509 g of 2-amino-4-chlorophenol (1.0512 mmol), 0.1304 g of pyrazinecarboxylic acid (1.0512 mmol), and 0.2617 g of dibutyltin oxide (0.8034 mmol). 0.3953 g (64%) dark brown solid; m.p. 207–209 °C (dec.); molar conductance, Λ_M_ (1 × 10^−3^ M, methanol): 23.5 ohm^−1^ cm^2^ mol^−1^ (non-electrolyte); UV-Vis [methanol, λ_max_/nm (ε/ M^−1^, cm^−1^)]: 219 (40728), 242 (40434), 268 (32905) π-π* (aromatic), 322 (22882) π-π* (C=N), 468 (19264) n-π* (C=N); IR (KBr) cm^−1^: 1639 *ν_asym_*(COO), 1584 *ν*(C=N), 1348 *ν_sym_*(COO), 1160 *ν*(C-O_Arom_), 544 *ν*(Sn-C), 513 *ν*(Sn-O), 416 *ν*(Sn-N); ^1^H NMR (300.52 MHz, CDCl_3_)δ: 0.51 (6H, t, *J* = 7.21 Hz, H-δ), 0.89-0.98 (8H, m, H-β, γ), 1.08-1.16 (4H, m, H-α), 7.00 (1H, d, *J* = 9.02 Hz, H-6), 7.26 (1H, dd, *J* = 2.40 Hz, *J* = 9.02 Hz, H-5), 7.56 (1H, d, *J* = 2.70 Hz, H-3), 7.66 (1H, ddd, *J* = 0.90 Hz, *J* = 5.10 Hz, *J* = 5.10 Hz, H-11), 7.84 (1H, d, *J* = 7.51 Hz, H-9), 8.11 (1H, dt, *J* = 1.50 Hz, *J* = 7.51 Hz, H-10), 8.82 (1H, s, ^3^*J*(^1^H-^119/117^Sn) = 14.42 Hz, H-7), 9.00 (1H, d, *J* = 2.70 Hz, H-16), 9.46 (1H, s_Broad_, H-17), 9.74 (1H, s, H-15), 9.78 (1H, d, *J* = 4.20 Hz, H-12); ^13^C NMR (75.57 MHz, CDCl_3_)δ: 166.1 (C-13), 163.4 (C-1), 151.0 (C-12), 148.2 (C-15), 147.72 (C-8), 147.70 (C-16), 144.0 (C-14), 143.2 (C-7), 141.5 (C-17), 139.6 (C-10), 133.5 (C-4), 130.2 (C-6), 126.81 (C-9), 126.79 (C-11), 122.8 (C-2), 120.1 (C-3), 116.2 (C-5), 32.4 (C-α, ^1^*J*(^119/117^Sn-^13^C) = 1100.30, 1050.41 Hz), 27.7 (C-γ, ^3^*J*(^119^Sn-^13^C) = 46.85 Hz), 26.1 (C-β, ^2^*J*(^119^Sn-^13^C)= 185.90 Hz), 13.9 (C-δ, ^4^*J*(^119^Sn-^13^C) = 13.60 Hz); ^119^Sn NMR (112.07 MHz, CDCl_3_)δ: −430; MS: (FAB^+^) [*m/z*] 531 [M^+^- Bu] (7), 465 [M^+^- C_4_H_4_N_2_COO] (22), 351 [M^+^- C_4_H_4_N_2_COO-2Bu] (10); Anal. Calcd. for C_25_H_29_N_4_O_3_SnCl: C, 51.09; H, 4.97; N, 9.53. Found: C, 50.69; H, 4.94; N, 9.35.

#### 2.1.4. 5,5-Di-n-butyl-10-nitro-1,4-diaza-6-oxatetracyclo [7.12.0.0^3,8^0^3,4^]-5-spirostanna-20,23-diaza-17-oxabyciclo-19,20,21-triene [0.0.0^19,20^] heptadeca-3,7,9,11,13,15-hexaene (1d)

Compound **1d** was prepared from 0.1126 g of 2-pyridinecarboxaldehyde (1.0512 mmol), 0.1620 g of 2-amino-4-nitrophenol (1.0512 mmol), 0.1304 g of pyrazinecarboxylic acid (1.0512 mmol) and, 0.2617 g of dibutyltin oxide (0.8034 mmol). 0.5141 g (82%) light orange solid; m.p. 190–192 °C (dec.); molar conductance, Λ_M_ (1 × 10^−3^ M, methanol): 9.9 ohm^−1^ cm^2^ mol^−1^ (non-electrolyte); UV-Vis [methanol, λ_max_/nm (ε/ M^−1^, cm^−1^)]: 216 (19997), 269 (25522) π-π* (aromatic), 320 (15695) π-π* (C=N), 433 (13299) n-π* (C=N); IR (KBr) cm^−1^: 1643 *ν_asym_*(COO), 1584 *ν*(C=N), 1355 *ν_sym_*(COO), 1162 *ν*(C-O_Arom_), 554 *ν*(Sn-C), 497 *ν*(Sn-O), 416 *ν*(Sn-N); ^1^H NMR (300.52 MHz, CDCl_3_)δ: 0.51 (6H, t, *J* = 7.21 Hz, H-δ), 0.91-0.97 (8H, m, H-β, γ), 1.05-1.16 (4H, m, H-α), 7.04 (1H, d, *J* = 9.31 Hz, H-6), 7.77 (1H, ddd, *J* = 0.90 Hz, *J* = 5.10 Hz, *J* = 5.10 Hz, H-11), 8.05 (1H, d, *J* = 7.51 Hz, H-9), 8.19 (1H, dd, *J* = 1.50 Hz, *J* = 7.81 Hz, H-10), 8.25 (1H, dd, *J* = 2.70 Hz, *J* = 9.62 Hz, H-5), 8.69 (1H, d, *J* = 2.70 Hz, H-3), 9.17 (1H, s, ^3^*J*(^1^H-^119/117^Sn) = 16.00 Hz, H-7), 9.08 (1H, d, *J* = 2.70 Hz, H-16), 9.48 (1H, s_Broad_, H-17), 9.77 (1H, s, *J* = 0.90 Hz, H-15), 9.84 (1H, d, *J* = 4.51 Hz, H-12); ^13^C NMR (75.57 MHz, CDCl_3_)δ: 165.7 (C-13), 170.1 (C-1), 151.2 (C-12), 148.5 (C-15), 148.0 (C-16), 147.3 (C-8), 146.6 (C-7), 143.5 (C-14), 141.0 (C-17), 140.2 (C-10), 136.0 (C-4), 129.3 (C-1), 128.6 (C-5), 127.9 (C-9), 127.7 (C-11), 121.2 (C-6), 114.0 (C-3), 32.4 (C-α, ^1^*J*(^119/117^Sn-^13^C) = 1153.19, 1123.72 Hz), 26.1 (C-β, ^2^*J*(^119^Sn-^13^C) = 194.97 Hz), 27.7 (C-γ, ^3^*J*(^119^Sn-^13^C) = 47.61 Hz), 13.3 (C-δ, ^4^*J*(^119^Sn-^13^C) = 14.36 Hz); ^119^Sn NMR (112.07 MHz, CDCl_3_)δ: −438; MS: (FAB^+^) [*m/z*] (%): 542 [M^+^- Bu] (7), 476 [M^+^- C_4_H_4_N_2_COO] (20), 362 [M^+^- C_4_H_4_N_2_COO- 2Bu] (8); Anal. Calcd. for C_25_H_29_N_5_O_5_Sn: C, 50.19; H, 4.89; N, 11.71. Found: C, 50.14; H, 4.88; N, 11.73.

### 2.2. Films and Devices Fabrication

Thin films and devices were made by spin-coating and vacuum thermal evaporation techniques. To monitor and characterize the films that integrate the devices, thin films of the same heptacoordinated organotin (IV) complexes were simultaneously deposited over monocrystalline silicon substrates (1 0 0), quartz y Corning glass. The films deposited using spin-coating technique was held in a Smart Coater 200 equipment at an angular speed of 330 rpm for 9 s. After deposition, the films were dried at 75 °C during 90 s on a hot plate. The vacuum thermal evaporation process was performed using a thermic evaporation equipment with a tantalum boat and two evaporation ports. The evaporation rate (organotin complexes ~48 Å/s, ZnPcF_16_ ~105 Å/s), temperature (298 K) and pressure (1 × 10^−6^ Torr) in the vacuum chamber were the same for all the deposition processes. Before deposition and except for monocrystalline silicon, the substrates were cleansed under an ultrasonic process using organic solvents (chloroform, methanol, and acetone) and dried with a Milwaukee 8975-6 dual temperature heat gun to guarantee an accurate performance of the devices. The silicon substrates were washed with “p” solution (10 mL HF, 15 mL HNO_3_ and 300 mL H_2_O), to remove surface oxide. The organotin complexes **1a–d** (Figure 1) were used to fabricate the electronic devices: **1a** (R=H), **1b** (R=Me), **1c** (R=Cl) and **1d** (R = NO_2_), on transparent substrate: coated glass with a tin-doped In_2_O_3_ (ITO) film.

## 3. Results and Discussion

### 3.1. Synthesis and Characterization of Heptacoordinated Organotin (IV) Complexes Complexes 1a–d

The heptacoordinated tin complexes 1a–1d were obtained by a multicomponent reaction, between 2-pyridinecarboxaldehyde, the 2-amino-4-R-phenol (R=H, CH_3_, Cl, NO_2_), dibutyltin oxide (IV), pyrazinecarboxylic acid and methanol as solvent in a stochiometric ratio as described in Figure 1. The resulting complexes were obtained in yields from 64% to 82%, as orange or dark red solids which were soluble in organic solvents as: chloroform, dichlorometane, methanol ethanol, and DMSO, with melting points in the range of 200 to 220 °C. The multicomponent strategy for the synthesis of the organotin(IV) compounds was selected taking into account our previous experiences, in general, the reaction proceeds in high yields and the mixtures of products that are difficult to purify were avoided. It was not necessary to prepare and characterize the ligand and the reaction time decrease in comparison to a two-step reaction [49,50]. The molar conductivity was analyzed in methanol solution, and the low values (9.9–31.8 ohm^−1^ cm^2^ mol^−1^) obtained indicate their non-electrolytic nature.

**Figure 2:**
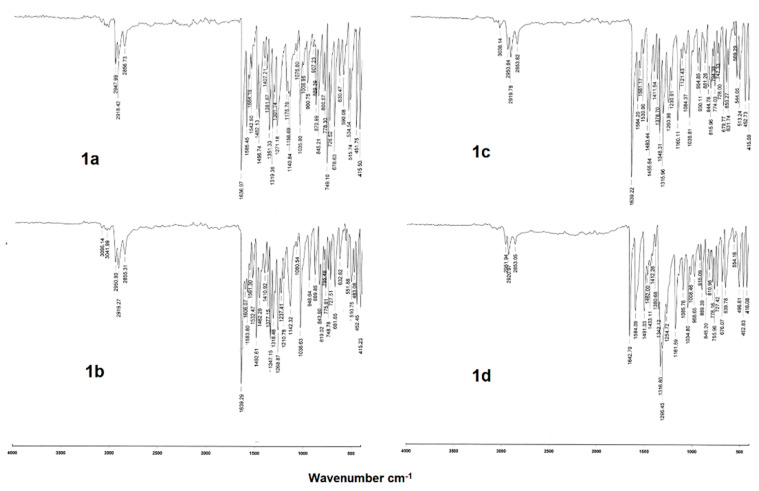
IR spectra of heptacoordinated organotin (IV) complexes (KBr).

The UV-vis spectra of the organotin (IV) complexes in Figure S1 (Supplementary Materials) were recorded in methanol solution (1 × 10^−3^ M). Observed bands in the region of 268 nm to 270 nm were assigned to the π-π* transitions, meanwhile, the bands at 322–336 nm and 433–379 nm to the π-π* and n-π* transitions, both associated with the azomethine group [51]. The spectral analysis showed a red shift in the absorption wavelength, due to the electron withdrawing effect of substituent on the aromatic ring. The IR spectroscopy provided evidence of the molecular structure of complexes **1a–1d**. The spectra in Figure 2 showed the vibration band ν(C=N) at 1585 cm^−1^ confirming the formation of the imine. The appearance of the vibration band ν(C–O) at 1141–1162 cm^−1^ demonstrated the coordination of the ligand, due to the deprotonation of the phenol and its consequent coordination to the tin atom. Additionally, the absence of vibrational bands associated with the carboxylic acid was indicative of the formation of the carboxylate and its coordination to the metallic center. The formation of the Sn-O bond was supported by the presence of two strong vibrational bands with two different absorptions in the range of 1347 cm^−1^ to 1643 cm^−1^, which correspond to the symmetric ν_sym_(COO) and asymmetric ν_asym_(COO) stretching vibrational modes of the carboxyl group. The energy difference between the asymmetric and symmetric carboxyl stretching vibrations Δν (COO) = (ν_asym_ − ν_sym_) provide valuable evidence of the Sn-carboxyl coordination mode. For complexes **1a–1d** the Δν calculated values are ranging from 286 to 326 cm^−1^. A Δν (COO) > 200 cm^−1^ is attributable to the formation of a covalent metal oxygen bond and a monodentate coordination mode of the carboxylic group bonded to the tin atom (see Table S1) [52,53]. The coordination of both lone pair of the nitrogen atoms from pyridine and azomethine to the tin was confirmed by the existence of a vibration band at 415 cm^−1^, corresponding to Sn-N. The expected band for Sn-O was observed around 513 cm^−1^.

The mass spectrometry for complexes **1a–1d** was performed using fast-atom bombardment (FAB^+^) ionization technique. The spectra showed that the most abundant peak corresponds to [M^+^-Bu]^+^ ion, confirming the formation of mononuclear organotin (IV) species (Figures S2–S5). All complexes exhibit analogous fragmentation pattern, fragment ions that involve cleavage of the carboxylate [M^+^− C_4_H_4_N_2_COO]^+^ or the carboxylate and two butyl groups bonded to the tin atom [M^+^ − C_4_H_4_N_2_COO − 2Bu]^+^ were identified. In none of the cases, the molecular ion was observed probably due to its instability and fast fragmentation, as has been reported for organotin (IV) complexes derived from pyridinecarboxylates [52,54].

**Figure 3:**
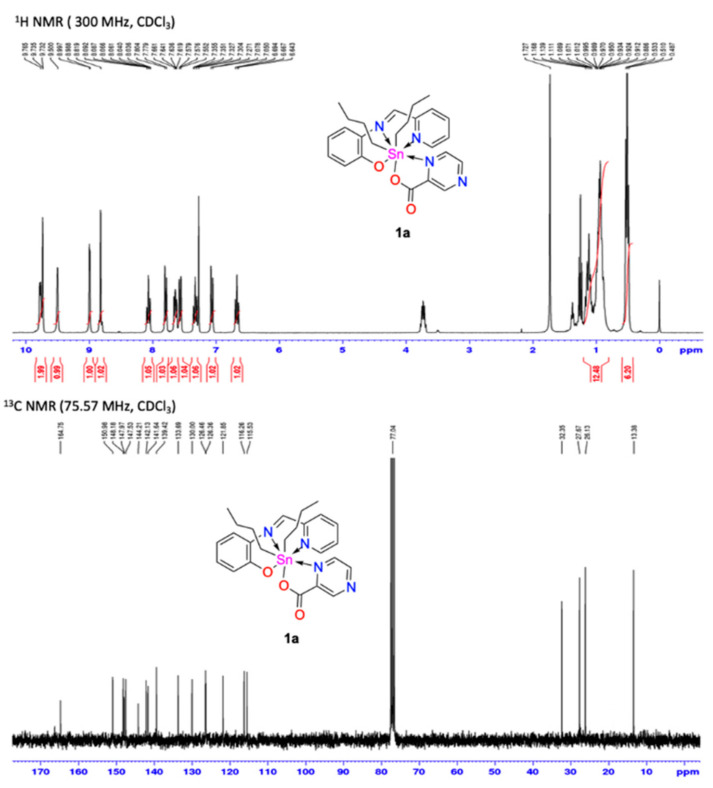
^1^H and ^13^C NMR of complex **1a** in CDCl_3_ solution.

The ^1^H, ^13^C and ^119^Sn NMR were acquired in CDCl_3_ solution. The ^1^H spectra showed the signals and multiplicity expected for each complex, the absence of the signals for the OH of the phenol and the caboxylic acid corroborated the deprotonation and formation of Sn–O bonds. The single signal H–7 of the azomethine (N=CH) was identified in the range of 9.2 to 8.8 ppm, and its satellite signals were observed due to the spin-spin coupling *^3^J* (^1^H-^119^Sn), indicative of the electron density transfer from the nitrogen of the imine group to the tin, and the presence of Sn-N coordination bond in solution. In the aromatic region, the signals for the pyridine, pyrazine and phenoxy rings were clearly identified and assigned. Additionally, the protons of the methyl of the butyl group attached to the tin exhibits a triple signal around 0.5 ppm. Figure 3 shows the spectra of complex **1a** as representative example, Figures S6–S21 show 1D and 2D NMR spectra of **1b–1c**. Respect to ^13^C NMR, all complexes displayed the anticipated signals in the aromatic and aliphatic regions. The spectra showed the carbons of the butyl groups in the region of 13.3 to 32.4 ppm as can be seen in Figure 3, and their satellite signals allowed us to measure the coupling constants *^n^J*(^13^C-^119^Sn). These values provide important information to assess the geometry around the tin atom in solution. The *^1^J*(^13^C-^119^Sn) values were found in the range of 1100 to 1153 Hz, indicating a seven-coordination of the tin atom. Additionally, by using the Lockhart equation (*^1^J*(^13^C-^119^Sn) = 11.4θ − 875) and the coupling constants values, the C-Sn–C bond angles, ranging from 173 to 177, were calculated. The comparison of these values with those described in both, solution and solid state for heptacoordinate dibutyltin (IV) complexes derived from Schiff bases and pyridinedicarboxylates where the tin acquires a pentagonal bipyramid (BPT) geometry [52], suggests a similar geometry around the tin atom. Where butyl groups may occupy the axial positions and consequently, the tridentate ligand and the carboxylate the equatorial plane.

**Figure 4:**
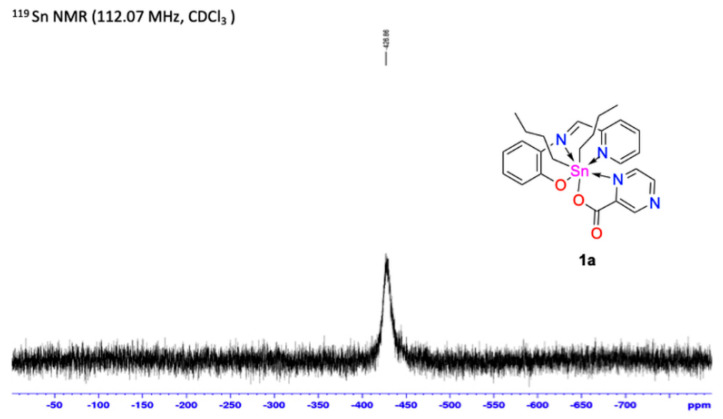
^119^Sn NMR of complex **1a** in CDCl_3_ solution.

The ^119^Sn NMR for complexes **1a–1d** was measured using a non-coordinated solvent (CDCl_3_) Figure 4 shows the **1a** spectrum. It is well known that chemical shifts values of organotin compounds are correlated with the coordination number around metallic center, in this case, a sharp single signal from –424 ppm to –430 ppm was observed. Which is an indicative of a heptacoordinated environment of the tin atom.

According to the structure of the heptacoordinated organotin (IV) complexes, these materials present conjugated molecules and are rich in π electrons. The latter is a sign of a semiconductor behavior, since the π orbitals in complexes can be energetically accessible for charges transport. It is expected that when a charge enters the complexes structure, it can be delocated over the π system, which would give place to a fast conduction in the complex molecule. But, even more important, the delocalization would also facilitate the charge transport between molecules, due to the better spatial overlapping of the delocated charge, with the adjacent molecules’ electronic states. Hence the importance of verifying the transport properties that show the heptacoordinated organotin (IV) complexes.

### 3.2. Manufacture and Characterization of Films and Optoelectronic Devices

**Figure 5:**
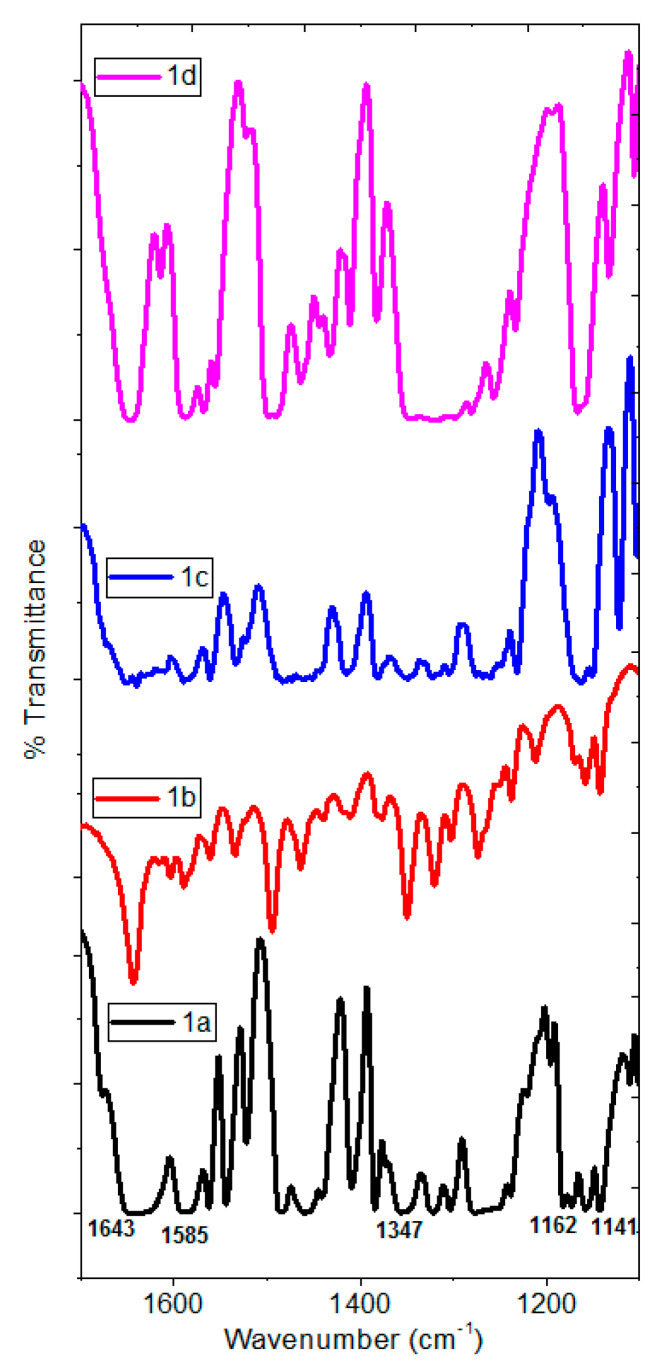
IR spectra of heptacoordinated organotin (IV) complexes in films.

**Figure 6:**
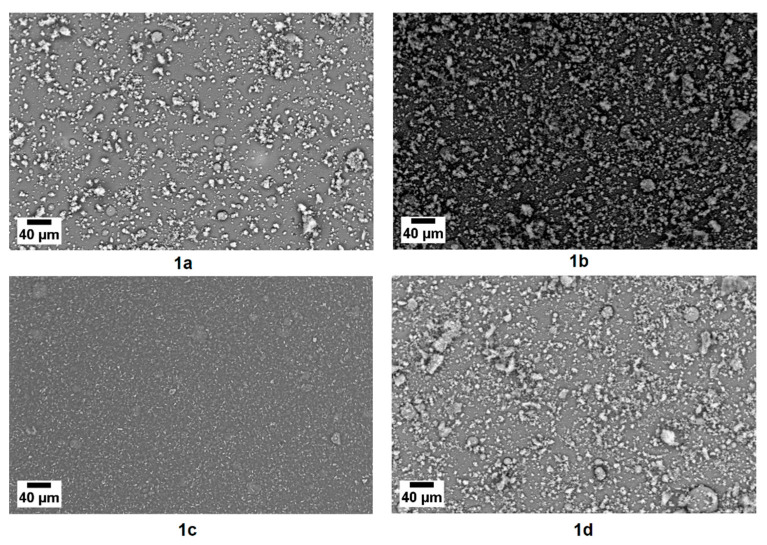
Microphotographs of films of heptacoordinated organotin (IV) complexes at 500×.

Heptacoordinated organotin (IV) complexes thin films were deposited to evaluate their stability and morphology. The chemical stability of the films manufactured by high vacuum evaporation was determined by means of IR spectroscopy, and the spectra obtained are shown in Figure 5. According to the analyzed results in the previous section for the synthetized complexes, the spectra show the vibration band ν(C=N) at 1585 cm^−1^, the vibration band ν(C–O) at 1141–1162 cm^−1^, and the two vibrational bands with two different absorptions in the range of 1347 cm^−1^ to 1643 cm^−1^, corresponding to the symmetric ν_sym_(COO) and asymmetric ν_asym_(COO) stretching vibrational modes of the carboxyl group. These results give an indication of the feasibility to manufacture thin films, with the heptacoordinated organotin (IV) complexes. It is important to consider that during the high vacuum evaporation, the complexes experiment phase changes: first, a change from solid to gas, and second, a change from gaseous state to the solid film, when the complex is deposited on the substrate. These thermal changes can degrade chemically the material, however in this case, the heptacoordinated organotin (IV) complexes show great stability. The use of this deposit technique allows to obtain high purity thin films, which may lead to high values of charge mobility. The efficient transport through the films of the heptacoordinated organotin (IV) complexes require that such films be continuous and without defects, such as fissures or holes. To verify the above, SEM was performed, and the microphotographs obtained are shown in Figure 6. Continuity along the film in the four films was observed. Also, a homogeneous particles distribution, that nucleated and grew in a second stage of the deposit, was observed. When starting the formation of the thin films, the complexes at a high temperature are deposited on the substrate that is at room temperature. This high thermal gradient leads, at the first place, to the formation of the film that completely covers the substrate. As the deposit progresses, the temperature in the material being deposited decreases, while the temperature of substrate (covered with the film of the complex) increases so, the thermal gradient decreases. This results in a new nucleus formation, which grow up to form particles of irregular shape. Considering that the conditions of deposit are the same for the four complexes, the difference in the shape of particles is due to the chemical composition of the individual complex and specifically, to the substituent. Also to its steric bulk and the conformation of the structure, that is affected by the size and the proximity of the molecular groups that compose each one of the four heptacoordinated organotin (IV) complexes.

According to SEM results, it would be expected that in the film of complex **1c** with the chloride substituent, which is the one with greater homogeneity and small size of particles, would show the best semiconductor behavior. To verify the above, the bandgap of both the pellet and film complexes was obtained from UV-vis spectroscopy. The bandgap or forbidden gap energy of the heptacoordinated organotin (IV) complexes was determined from the equation:

(1) $$E_{g}=\;\frac{hc}{\lambda_{g}}$$

where *E_g_* is the forbidden gap energy, h is the Planck constant, *c* is the speed of light and λ*_g_* is the wavelength associated to the forbidden gap energy. It is important to consider that the bandgap of the complexes embedded in the PEDOT:PSS matrix was also evaluated. All results are presented in Table 1, where the bandgap for the thin films is observed to be lower with respect to the pellets, due to their comparatively high charge-carrier mobility. Additionally, the values indicate that the defect concentration in the films is very low [55]. Although the gap values are very close to each other, it is the complex **1c** film that presents the smallest gap due to its greater homogeneity and smaller particle size (see Figure 6). On the other hand, when the particles of the heptacoordinated organotin (IV) complexes are embedded in the polymer, the gap increases considerably due to the bulk heterojunction of the complex-PEDOT: PSS system, however, the obtained values remain in the range of organic semiconductors. In the bulk heterojunction, both phases, the organotin (IV) complex and the polymer are intimately intermixed. This mixture has a priori no symmetry breaking in the volume. There is no preferred direction for the internal fields of separated charges; that is, the electrons and holes created within the volume have no net resulting direction in which they should move [56,57].

**Figure 7:**
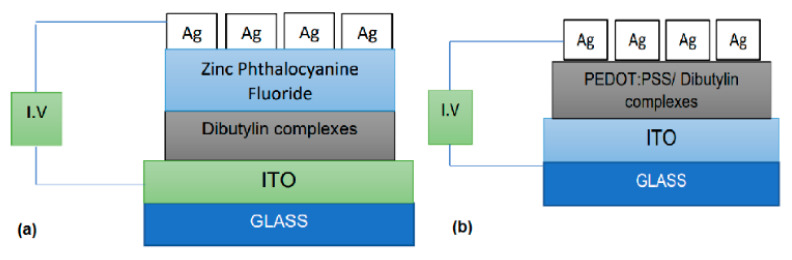
Scheme of the electronic devices manufactured by (**a**) evaporation and (**b**) spin-coating.

Devices with a “contact up/base down” configuration were manufactured by using the heptacoordinated organotin (IV) complexes **1a–d** films to compare the electrical characteristics among them, for electronic applications. As shown in Figure 7, two different devices were manufactured: glass/ITO/organotin (IV) complex/F_16_ZnPc/Ag and glass/ITO/PEDOT:PSS-organotin (IV) complex/Ag by evaporation and spin-coating respectively. The device of Figure 7a was manufactured with planar heterojunction, while the device of Figure 7b was manufactured with bulk heterojunction architecture. Figure 7a films thicknesses are of 22.9, 15.8, 81.3 and 9.4 nm for complex 1a, 1b, 1c, 1d respectively. Moreover, the total films thicknesses are of 113.6, 43.6, 108.2 and 122.6 nm for complex 1a, 1b, 1c, 1d evaporated devices respectively (Figure 7a) and 50 nm for all the spin-coated devices (Figure 7b). Whereas for the devices surface, 6.25 cm^2^ was maintained for all the manufactured devices. Regarding the device operation, where both charges positive and negative are injected from the electrodes, it is important that the materials through which these charges are transported should have an optimal alignment of their energy levels. Thus, it is desirable that the layers through which the holes are transported within the device, have similar HOMO (highest occupied molecular orbital) energy. Due to the previous, Figure 7b device PEDOT:PSS with a −5.2 eV HOMO, acts as a hole carrier layer toward the ITO anode with a 4.7 eV work function. Conversely, for the Figure 7a device, the F_16_ZnPc film with a −3.4 eV LUMO (lowest unoccupied molecular orbital) through which the electrons travel, is in contact with the silver cathode, with a 4.2 eV work function. This fact will lead to an efficient charge transfer between the cathode and the device internal layers, by reducing the energy barriers derived on the electrons transfer and facilitating an electric current conduction in the organotin (IV) complexes, as active layer.

**Figure 8:**
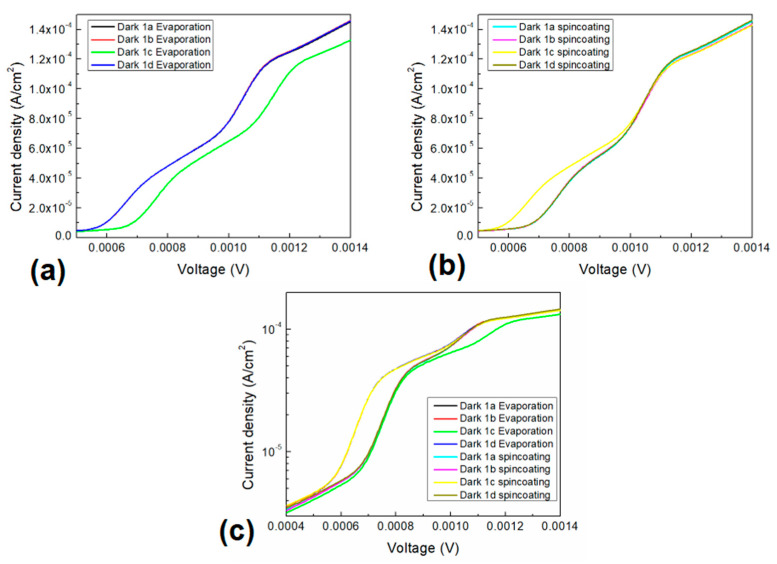
J-V characteristic for the (**a**) evaporated and (**b**) spin-coated and (**c**) semilogarithmic J-V characteristic for the organotin (IV) complexes devices.

The dark J-V characteristics for the evaporated and spin-coated devices are shown in Figure 8a,b, correspondingly. The charge transport parameters for the devices were obtained by analyzing the forward dark current density as a function of the applied voltage (J-V). The selected voltage range was intended to not damage the device during measurement, which allows us to evaluate their characteristics for low voltage applications. The devices electrical behavior shows a nonlinear curve that resembles a Schottky curve at low voltages (<1.5 mV). Moreover, a current density as large as ~1.4 × 10^−4^ A/cm^2^ at a fixed voltage of ~1.4 mV has been observed. Figure 8a shows a variation of the curve only for the organotin (IV) complex **1c**, the rest of the devices present a similar curve. It can be observed the **1c** curve start to increase its current density for values higher than ~0.7 mV while for the rest of the devices at ~0.6 mV. Also, the **1c** device curve shape presents an interesting variation compared to the other devices and a current density variation of as much as ~3 × 10^−5^ A/cm^2^ at ~1.1 mV can be observe. The latter could be related to the previously observed small sized particles that may affect the conduction mechanisms of the films. On the other hand, Figure 8b shows a variation of the curve only for the organotin (IV) complex **1c**, the rest of the devices present a similar curve. Also, a slight variation of the curves can be observed for the devices for voltages greater than ~1.1 mV. It can be observed the **1c** curve starts to increase its current density for values higher than ~0.6 mV while for the rest of the devices at ~0.7 mV. Also, the **1c** device curve shape presents a small variation compared to the other devices for voltages higher than 1 mV and a larger variation for lower voltages. Where a current density variation of as much as ~2.9 × 10^−5^ A/cm^2^ at 0.7 mV can be observed. The difference in the electric behavior of the devices with the complex **1c** may be due to its lower bandgap, and the presence of the substituent. Substituents in the tin complex generate anisotropy in the active layers of the devices. Moreover, the chlorine group in complex **1c** attracts electrons by inductive effect and donates electrons by mesomeric effect, where the latter is greater than the inductive effect. Although, the methyl group in complex **1b** is an electron donor one, and the NO_2_ in complex **1d** is an attractor group. On other hand, comparing both device structures J-V characteristics, it should be mention that the current density values and shape of the curve for the spin-coated complex **1c** device is comparable to the evaporated complexes **1a**, **1b** and **1d** devices. Also, comparing both manufactured structures characteristics, it can be observed that the shape of the curve is stepper for most of the spin-coated devices than for the evaporated devices. Figure 8c shows the semilogarithmic J-V characteristic for all the manufactured organotin (IV) complexes devices. The variation previously observed for the different structures and the complexes are shown, but also a slight variation of the curves for voltages lower than 0.6 mV can be observed. Also, it is worth to mention that the current of the complex **1c** evaporated device for voltages higher than 1.2 mV and lower than 0.6 mV is slightly lower than the rest of the devices. Table 2 shows the organotin (IV) complex devices electrical parameters, such as the slope of the ohmic region, slope of the space charge limited current (SCLC) region, the carrier mobility at SCLC, the threshold voltage (Vth), the saturation current (Io) and the diode ideality factor (n) and the photocurrent at 0 V. The observed results indicate that the manufactured devices may function as a good optoelectronic device for low power applications.

The forward current through the Schottky junction was determined using the following expression:

(2) $$I=I_{s}\exp\left(\frac{\mathrm{qV}}{\mathrm{nkT}}\right)$$

where V is the applied voltage, I_s_ is the saturation current and n is the diode ideality factor. Additionally, for the organotin (IV) complex devices it can be divided into two conduction mechanism regions: ohmic and SCLC.

**Figure 9:**
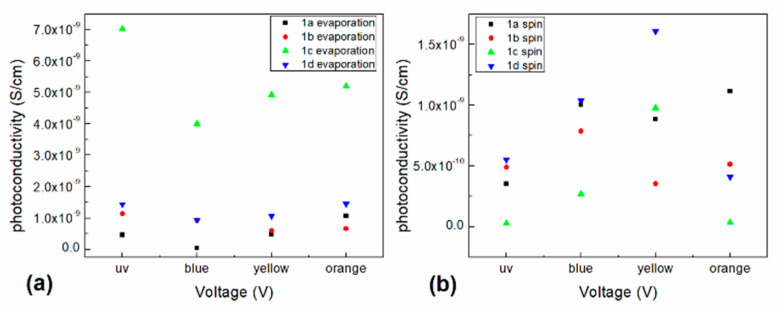
Incident light dependent photoconductivity for the (**a**) evaporated and (**b**) spin-coated organotin (IV) complexes devices.

First, it is worth to mention that the slopes of the ohmic region are close to 1 while for the SCLC are close to 2. Comparing the evaporated devices, the lowest ohmic region slope is observed for the complex 1d while the highest is observed for the complex 1c, with a variation of ~0.11. For the SCLC region the lowest slope is for the complex 1a and the largest for the complex 1c with a variation of ~0.06. On the other hand, for the spin-coated devices, in comparison to the previous, the slope values are higher for all the devices except for the complex 1c which become smaller for both, the ohmic and SCLC regions. The lowest ohmic region slope is observed for the complex 1c while the highest is observed for the complex 1b, with a variation of ~0.15. For the SCLC region the lowest slope is observed for the complex 1c and the largest for the complex 1a with a variation of ~0.33. The latter implies a change in the carrier mobility and therefore the conductivity of the organotin (IV) complex devices, for both manufactured structures. Additionally, the previously observed J-V characteristics for the complex 1c devices are strongly dependent on the mentioned behavior of the carrier mobility on the ohmic and SCLC regions. Moreover, evaluating the diode ideality factor, it can be observed that there is a small difference in n from the ideal diode (n=1) and the device operation is close to the ideal diode. Such variations may be related to no film homogeneity or interfaces quality. The highest n is observed for the evaporated complex 1c, while the lowest is observed for the spin-coated complex 1b. The saturation current was observed to lay between 1 × 10^−5^ A and 1.3 × 10^−5^ A, while the threshold voltage lay between 3.5 × 10^−4^ V and 4.8 × 10^−4^ V for the manufactured devices, which supports what is observed on Figure 8. The saturation current for the evaporated structures shows similar values except for the complex 1c and for the spin-coated structures, complex 1c is of similar value than those of the evaporated structures. Also, the lowest current is observed for the spin-coated complex 1b device structure. On the other hand, comparing the threshold voltage of the evaporated structures, the devices present similar values except for the complex 1c which is the largest. But for the spin-coated structures, the devices also present similar values except for the complex 1c which is the smallest, and with a threshold voltage comparable to those observed for the evaporated structures. The variation in behavior that occurs between devices with complexes 1b and 1c is interesting. The difference in the polarity of the substituents is the possible cause of these differences, since the methyl of complex 1b is non-polar, the chlorine of complex 1c has a high polarity. The current density for the SCLC region can be expressed as follows [56]:

(3) $$J_{SCLC}=\frac{9\varepsilon_{r}\varepsilon_{0}\mu V^{2}}{8L^{3}}$$

where *μ* is the mobility, L is the film thickness $\varepsilon_{r}$ and $\varepsilon_{0}$ are the relative material (2.2 for the PEDOT:PSS [57] and approximated to 1.56 for the ZnPc [48]) and vacuum (8.85 × 10^−14^ F cm^−1^) permittivity respectively. Table 2 contains the estimated mobility values for the films in the SCLC region. The organotin (IV) complex devices present a carrier mobility of ~10^−2^–10^−1^ cm^2^/Vs, where the highest mobility (0.86 cm^2^/Vs) was obtained for the evaporated complex 1d device that causes the higher conductivity for this particular device. The mobility of the spin-coated devices is very similar among them and most of them are lower compared to those of the evaporated devices, consequence of the conduction mechanisms derived from the interaction with PEDOT:PSS matrix. The obtained mobility values are quite large compared to similar materials [58], which can be attributed to defect free films and homogeneity that implies no carrier trapping behavior. Furthermore, I-V characteristics were conducted in both darkness and illumination conditions and the observed photocurrent at 0 V was obtained for the manufactured devices and shown in Table 2. It can be observed that the generated photocurrent was of approximately 10^−7^ A for all the manufactured structures, which indicates that the devices present a small photovoltaic effect induced by the illumination. For the evaporated devices, it is observed that the highest photocurrent was obtained for the complex 1c and the rest present the following behavior 1c > 1d > 1b > 1a. Whereas for the spin-coated devices the highest photocurrent was obtained for the complex 1b and the rest present the following behavior 1b > 1c > 1a > 1d. Further evaluation of the optoelectronic devices under different incident light colors was conducted. Consequently, the photoconductivity for the evaporated and spin-coated organotin (IV) complex devices was calculated and plotted on Figure 9. This figure shows a light dependent variation of the photoconductivity for both structures. It is worth to mention that most of the shown photoconductivity values lay between 0 V and 1.7 × 10^−9^ S/cm, which are quite small, except for the evaporated complex 1c device which presents a photoconductivity between 4 × 10^−9^ and 7 × 10^−9^ S/cm. For the evaporated complex devices, a similar behavior of the photoconductivity with the different incident light colors is observed. The lowest photoconductivity was observed for the blue incident light for most of the complex devices, except for complex 1c which presents its lowest value for the yellow incident light. Most of the photoconductivity values have the trend which follows the order by the complex 1c >1d > 1b > 1a. Moreover, the spin-coated complex devices present no specific trend, but complexes 1c and 1d show similar behavior while 1a and 1b show also similar behavior with the incident light colors. A variation of as large as approximately 3 × 10^−9^ S/cm and 1.2 × 10^−9^ S/cm was observed for the evaporated and spin-coated complex devices, respectively. Comparing both complex 1c devices, it is possible to observe a large effect of the device structure and manufacturing process. Where the F_16_ZnPc incorporation, may conduct to larger photoconductivity values as observed, consequence of its electron extraction capability and energy match with the silver contact workfunction. However, the main cause of the changes in photocurrent of the devices is the type of substituent in the organotin (IV) complexes.

## 4. Conclusions

Four mononuclear heptacoordinated organotin (IV) complexes were prepared by using a multicomponent reaction, the reaction proceeded in good yields. Thin films with high thermal stability were deposited, on which the bandgap was evaluated. The values obtained between 1.23 and 1.40 eV, place these complexes within the range of organic semiconductors. The lowest bandgap was obtained for the complex with the chloride substituent (complex 1c), this due to its high electron-attracting capacity. Moreover, the manufacture of a heptacoordinated organotin (IV) complexes-PEDOT:PSS-based composite optoelectronic device was conducted and compared with a different device architecture. Besides, complex 1c devices are strongly dependent on the carrier mobility on the ohmic and SCLC regions. A Schottky curve at low voltages (<1.5 mV) and a current density variation of as much as ~3 × 10^−5^ A/cm^2^ at ~1.1 mV was observed for complex **1c** device. A generated photocurrent was of approximately 10^−7^ A and a photoconductivity between 4 × 10^−9^ and 7 × 10^−9^ S/cm for all the manufactured structures. However, in complex **1c** devices it is possible to observe a large effect of the device structure and manufacturing process. A variation of as large as approximately 3 × 10^−9^ S/cm and 1.2 × 10^−9^ S/cm was observed for the evaporated and spin-coated complex devices. The chlorine incorporation in **1c** serves as a source of negative fixed charge and also reduces the band gap. This research opens new opportunities to explore the potential of organotin (IV) compounds in the design of novel devices with attractive electronic and optoelectronic properties at low cost. Organotin (IV) compounds can provide a plethora of molecular architectures and are useful building blocks due to the ease of the synthesis. The feasibility to modify the π-conjugation by the incorporation of several functional groups, provides unique chemical and physical characteristics that result in the modification of the thin films energy bandgap. Finally, the incorporation of the heptacoordinated organotin (IV) complexes within the PEDOT:PSS generated a bulk heterojunction that can be applied in the fabrication of devices with specific applications like OLEDs, photodetectors and chemical/bio sensors.

## Figures and Tables

**Table 1:** Forbidden gap energy of heptacoordinated organotin (IV) complexes.

Sample	E_g_ (eV) Pellet	E_g_ (eV) Evaporated Film	E_g_ (eV) Complex-PEDOT:PSS Film
1a	1.98	1.37	3.17
1b	1.89	1.40	3.27
1c	1.88	1.23	3.48
1d	1.95	1.31	3.10

**Table 2:** Organotin (IV) complexes devices electrical parameters.

Sample	Slope Region	Slope Region	Mobility	Vth	Is	n	Photocurrent
	1	2	cm^2^/V s	V	A		A
	Ohmic	SCLC					@ 0 V
Dark 1a Evaporation	0.812	2.015	6.84 × 10^−1^	3.59 × 10^−4^	1.29 × 10^−5^	1.06	1.29 × 10^−^^7^
Dark 1b Evaporation	0.809	2.027	3.89 × 10^−2^	3.61 × 10^−4^	1.29 × 10^−5^	1.12	1.52 × 10^−^^7^
Dark 1c Evaporation	0.918	2.079	5.27 × 10^−1^	4.32 × 10^−4^	1.09 × 10^−5^	1.53	2.98 × 10^−^^7^
Dark 1d Evaporation	0.804	2.020	8.63 × 10^−1^	3.59 × 10^−4^	1.30 × 10^−5^	1.11	1.68 × 10^−^^7^
Dark 1a Spincoating	0.910	2.321	4.38 × 10^−2^	4.79 × 10^−4^	1.18 × 10^−5^	1.32	1.97 × 10^−^^7^
Dark 1b Spincoating	0.955	2.274	4.34 × 10^−2^	4.76 × 10^−4^	1.06 × 10^−5^	0.68	2.99 × 10^−^^7^
Dark 1c Spincoating	0.809	1.999	4.08 × 10^−2^	3.52 × 10^−4^	1.30 × 10^−5^	1.11	2.81 × 10^−7^
Dark 1d Spincoating	0.900	2.307	4.38 × 10^−2^	4.78 × 10^−4^	1.23 × 10^−5^	1.23	9.83 × 10^−8^

## Data Availability

Data is contained within the article or Supplementary Materials.

## Supplementary Materials

The following are available online at https://www.mdpi.com/2073-4360/13/7/1023/s1, Figure S1: UV-vis absarbance in methanol of 1a-1c, Figure S2: Mass spectrum FAB^+^ of 1a, Figure S3: Mass spectrum FAB^+^ of 1b, Figure S4: Mass spectrum FAB^+^ of 1c, Figure S5: Mass spectrum FAB^+^ of 1d, Figure S6: COSY spectrum of 1a, Figure S7: HSQC spectrum of 1a, Figure S8: HMBC spectrum for 1a, Figure S9: 1H NMR spectrum of 1b, Figure S10. 13C NMR spectrum of 1b, Figure S11: COSY spectrum of 1b, Figure S12: HSQCspectrum of 1b, Figure S13: HMBC spectrum of 1b, Figure S14: 1H NMR spectrum of 1c, Figure S15: 13C NMR spectrum of 1c, Figure S16: COSY spectrum of 1c, Figure S17: HSQC spectrum of 1c, Figure S18: HMBC spectrum of 1c, Figure S19: ^1^H NMR spectrum of 1d, Figure S20: 13C NMR spectrum of 1d, Figure S21: COSY spectrum of 1d, Figure S22: HMBC spectrum of 1d, Figure S23: 119Sn NMR in CDCl3 solution of 1b, Table S1: Characteristic IR stretching frequencies of carboxylate group.
