# Supplementary material for: Design of Promising Heptacoordinated Organotin (IV) Complexes-PEDOT: PSS-Based Composite for New-Generation Optoelectronic Devices Applications
Source: Polymers (Basel). 2021 Mar 25;13(7):1023. doi: 10.3390/polym13071023 (PMC8038072; doi:10.3390/polym13071023)
Supplement: Supplementary file 1 [file polymers-13-01023-s001.pdf]

Supplementary Material

Design of Promising Heptacoordinated Organotin (IV) Complexes-PEDOT: PSS-Based Composite for New-Generation Optoelectronic Devices Applications

María Elena Sánchez-Vergara[1], Leon Hamui[1*], Elizabeth Gómez[2*], Guillermo M. Chans[2] and José Miguel Galván-Hidalgo[2]

[1] Facultad de Ingeniería, Universidad Anáhuac México, Avenida Universidad Anáhuac 46, Col. Lomas Anáhuac, Huixquilucan 52786, Estado de México, México;
[2] Instituto de Química, Universidad Nacional Autónoma de México, Circuito Exterior s/n. C.U., Delegación Coyoacán, C.P. 04510. Ciudad de México, México;
*Correspondence: leon.hamui@anahuac.mx (L.H.), eligom@iquimica.unam.mx (E.G.)

Table S1. Characteristic IR stretching frequencies of carboxylate group.

Compound	ν_{asym}(COO)	ν_{sym}(COO)	$\Delta\nu$ (COO)
1a	1637	1351	286
1b	1639	1347	292
1c	1639	1348	291
1d	1643	1355	288

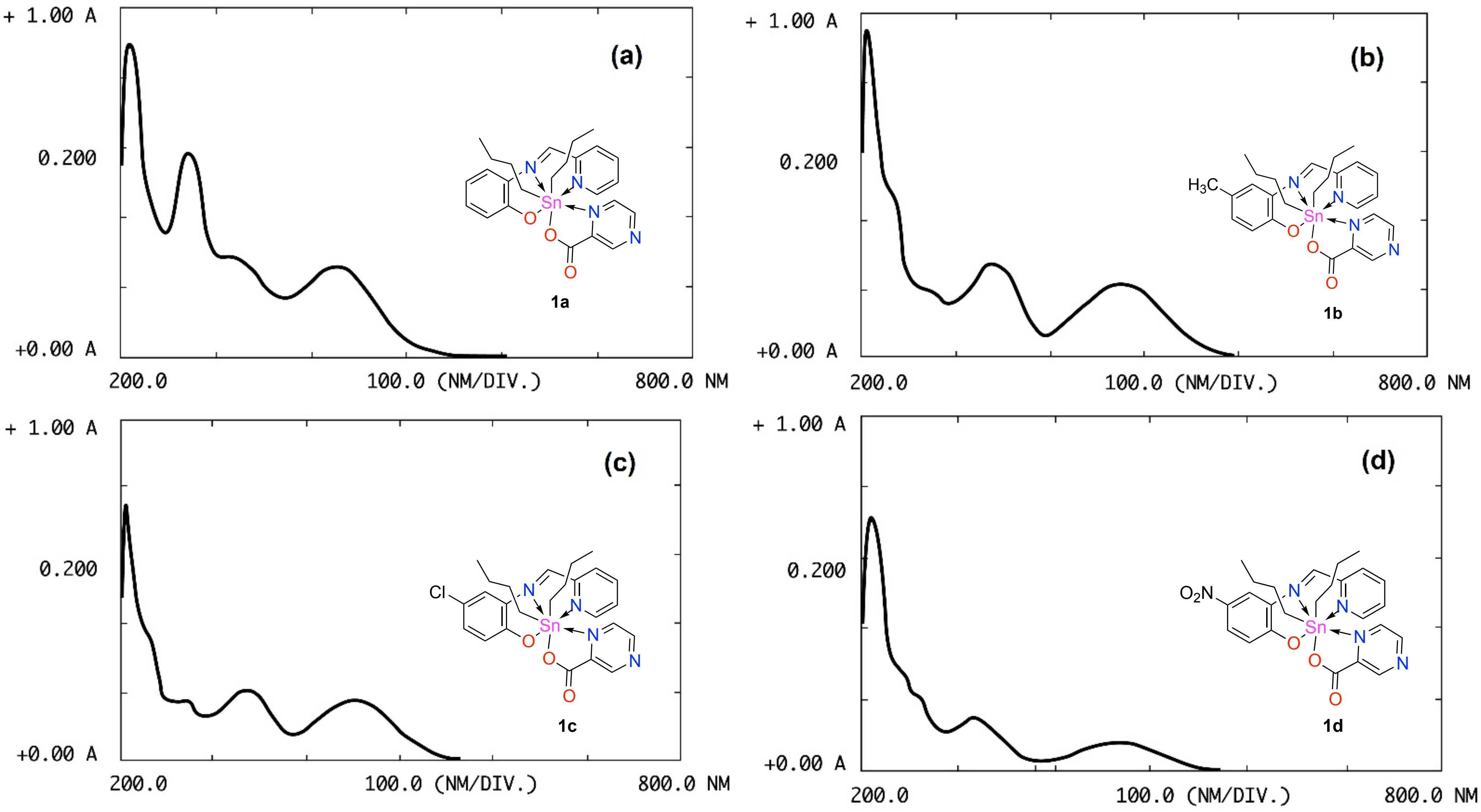

Figure S1. UV-vis absarbance in methanol of **1a**-**1c**.

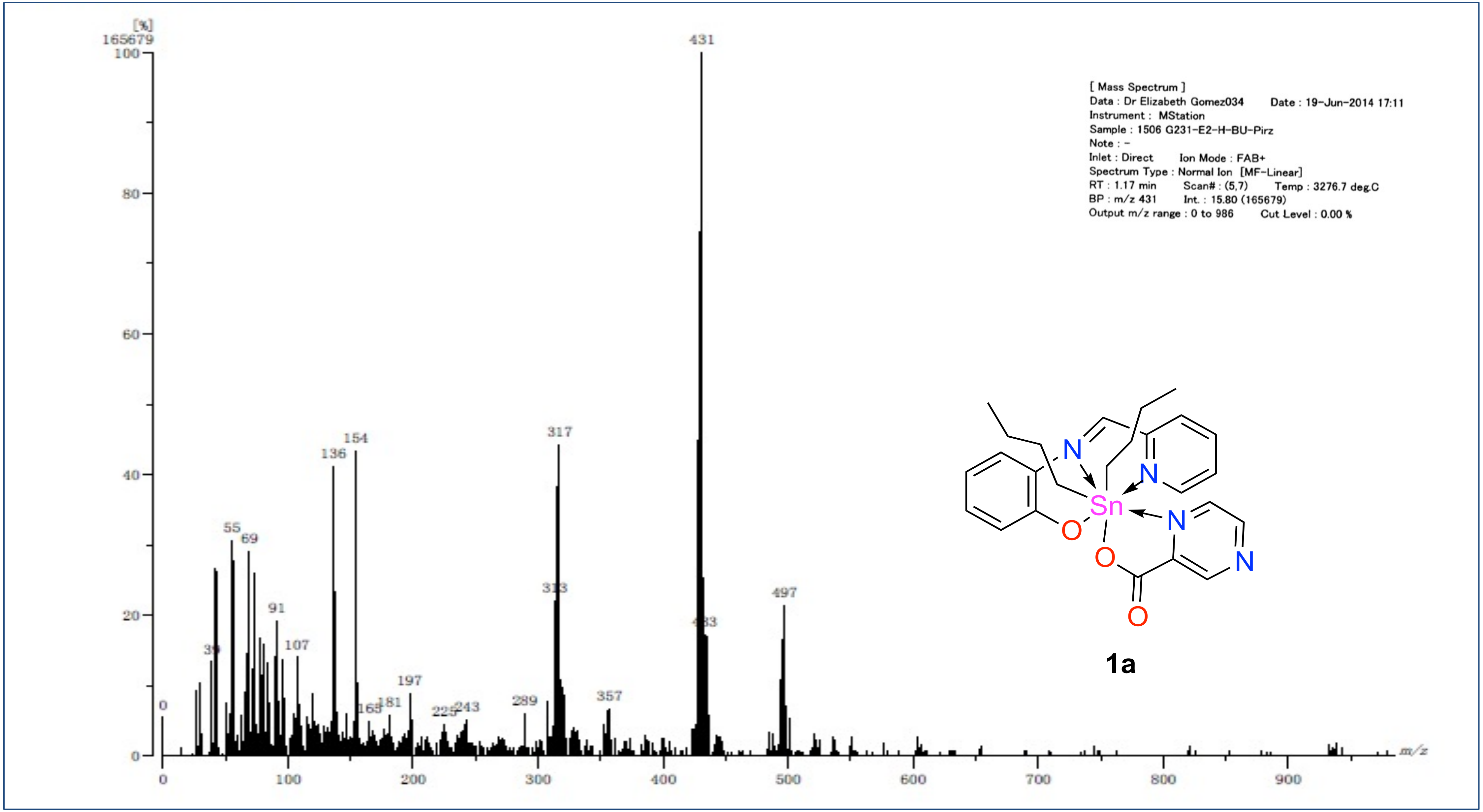

Figure S2. Mass spectrum FAB+ of **1a**.

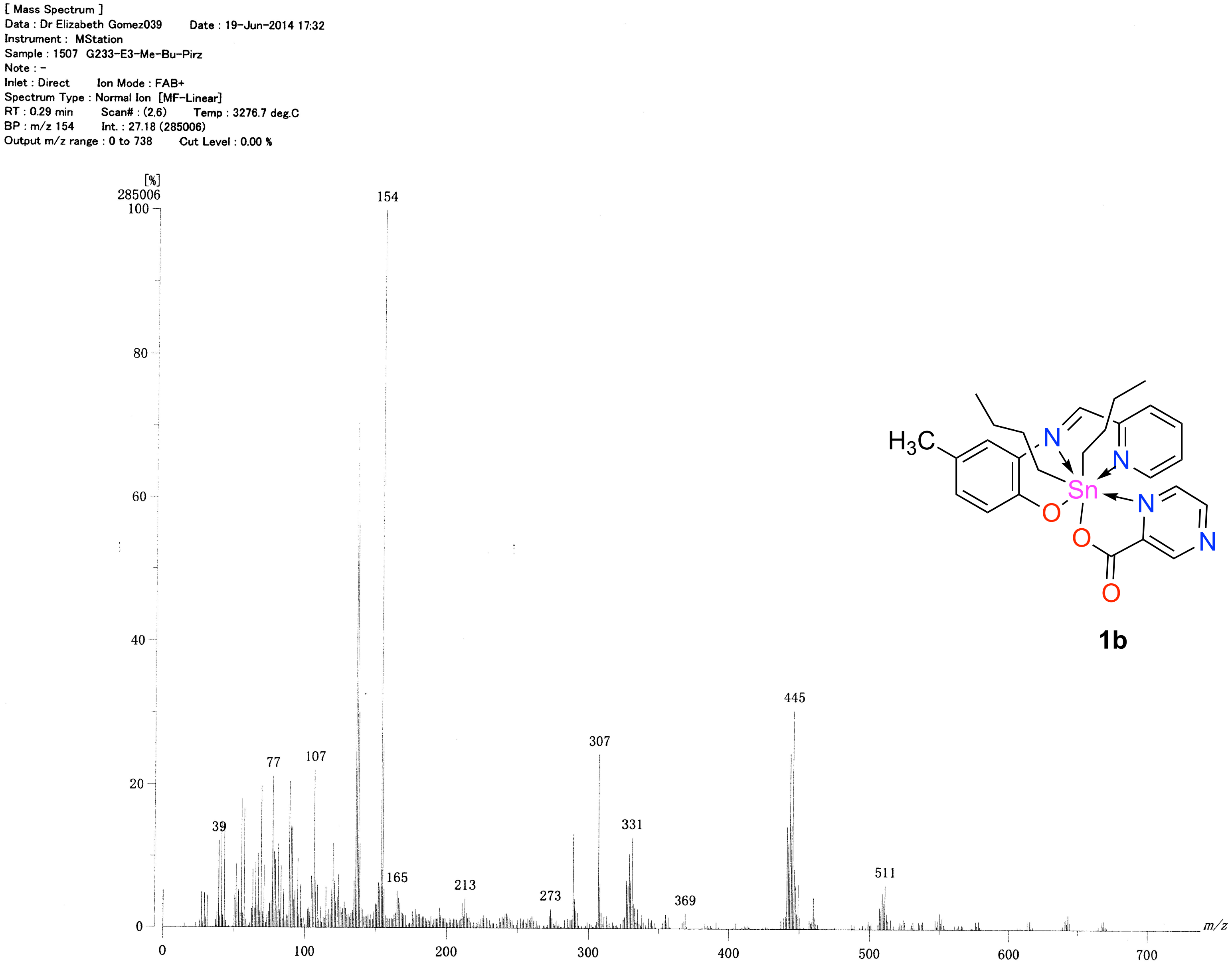

Figure S3. Mass spectrum FAB+of **1b**.

[Mass Spectrum]
Data : Dr Elizabeth Gomez041 Date : 19-Jun-2014 17:41
Instrument : MStation
Sample : 1509 G235-E5-Cl-Bu-Pirz
Note : -
Inlet : Direct Ion Mode : FAB+
Spectrum Type : Normal Ion [MF-Linear]
RT : 0.88 min Scan# : (4,17) Temp : 3276.7 deg.C
BP : m/z 154 Int. : 39.40 (413111)
Output m/z range : 0 to 698 Cut Level : 0.00 %

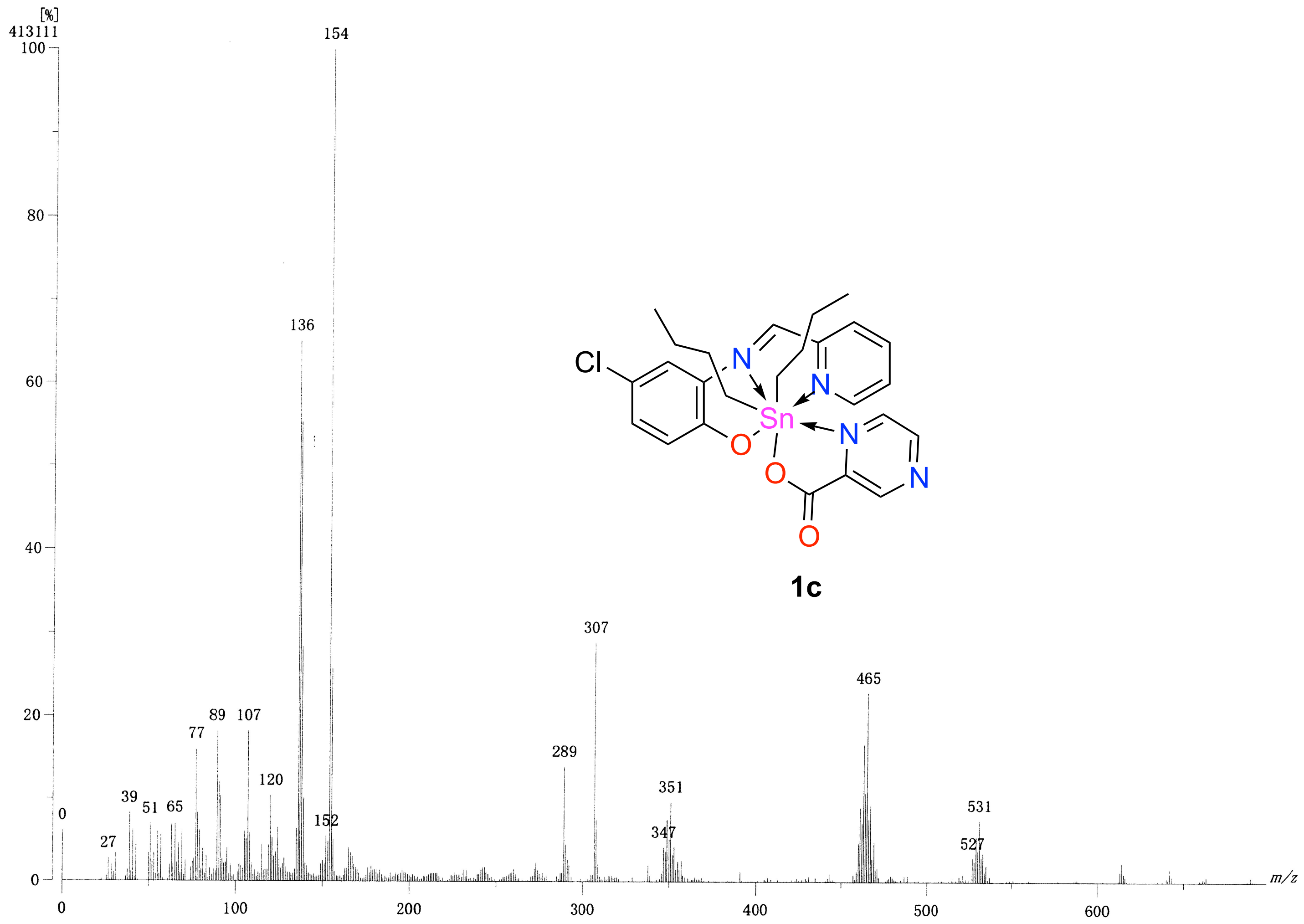

Figure S4. Mass spectrum FAB$^+$ of **1c**.

[Mass Spectrum]
Data : Dr Elizabeth Gomez040 Date : 19-Jun-2014 17:35
Instrument : MStation
Sample : 1508 G234-E4-NO2-Bu-Pirz
Note : -
Inlet : Direct Ion Mode : FAB+
Spectrum Type : Normal Ion [MF-Linear]
RT : 0.29 min Scan# : (2,10) Temp : 3276.7 deg.C
BP : m/z 154 Int. : 17.35 (181913)
Output m/z range : 0 to 684 Cut Level : 0.00 %

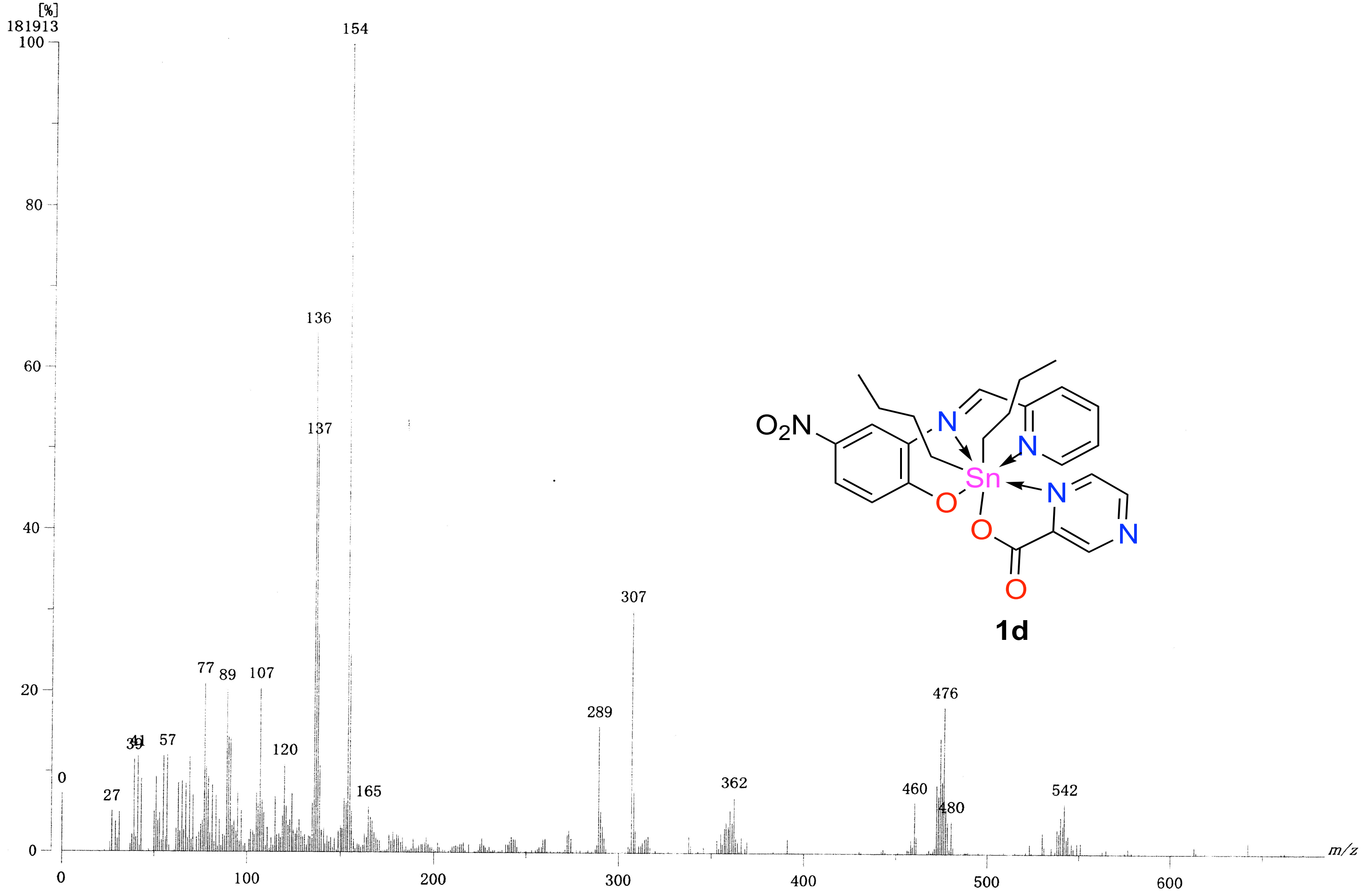

Figure S5. Mass spectrum FAB$^+$ of **1d**.

G231-E2-H-Bu-Pirz
COSY

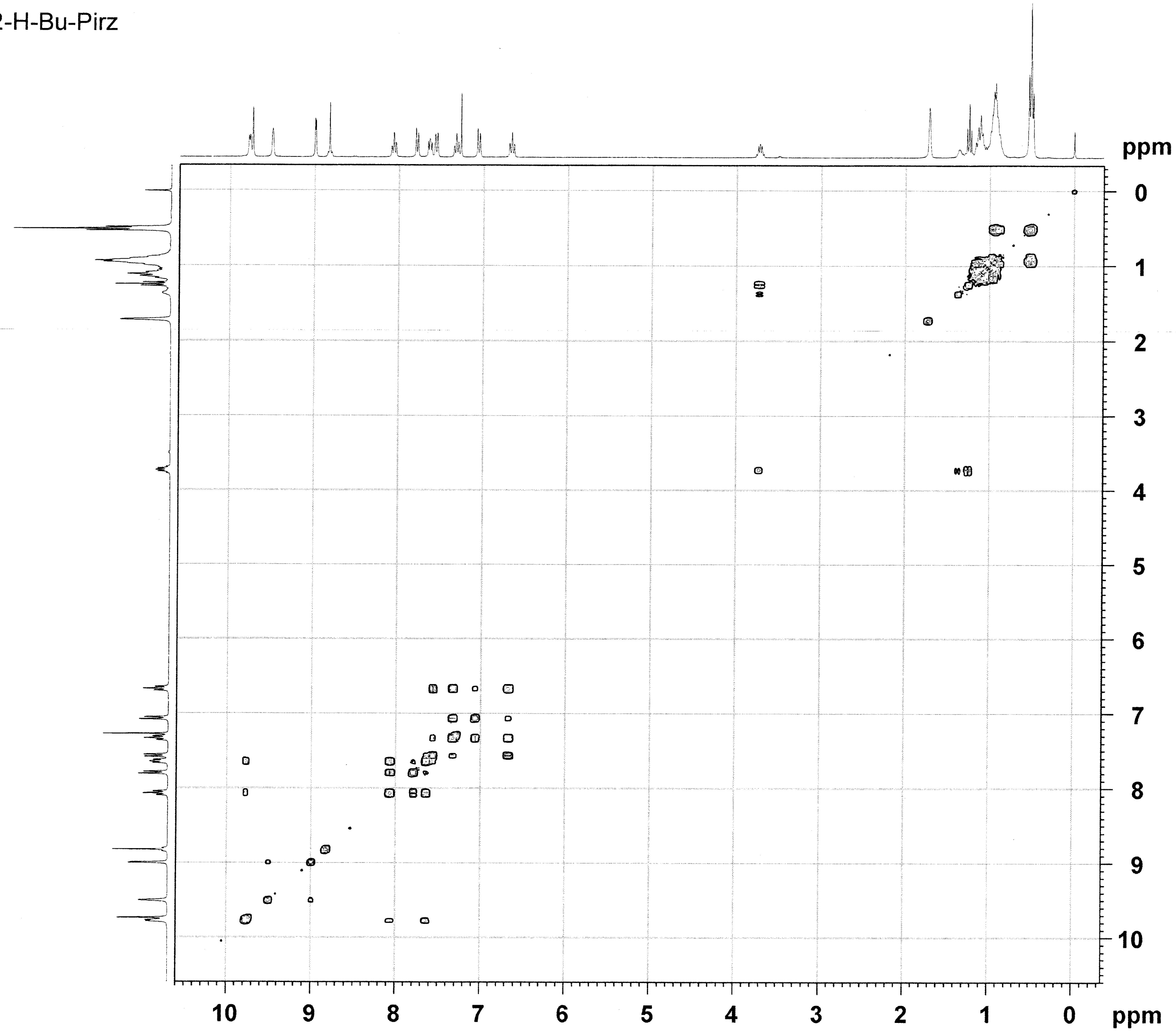

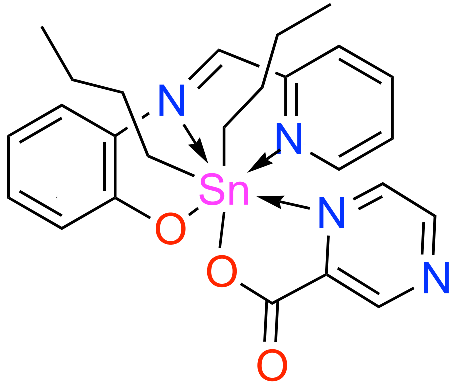

1a

Figure S6. COSY spectrum of **1a**.

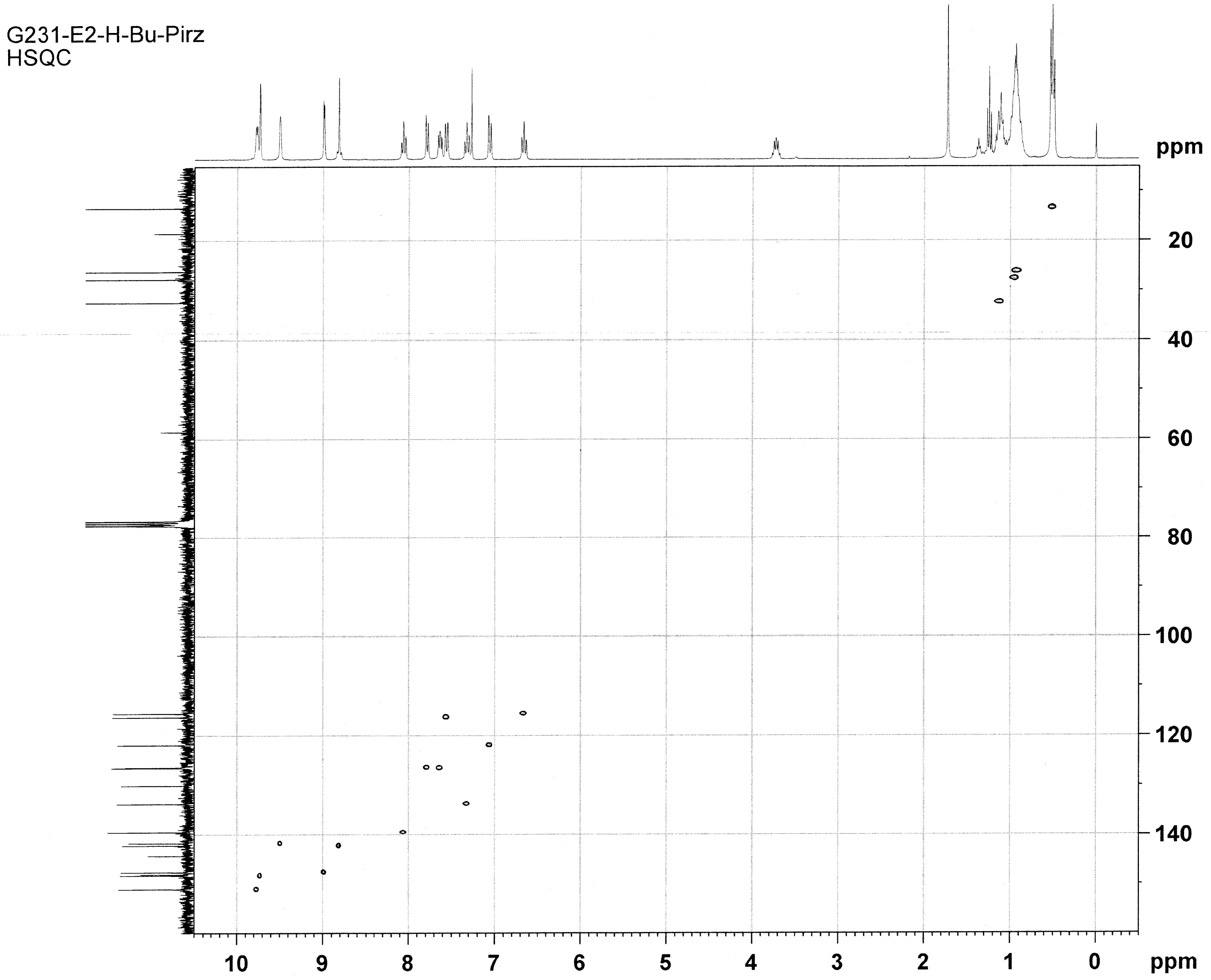

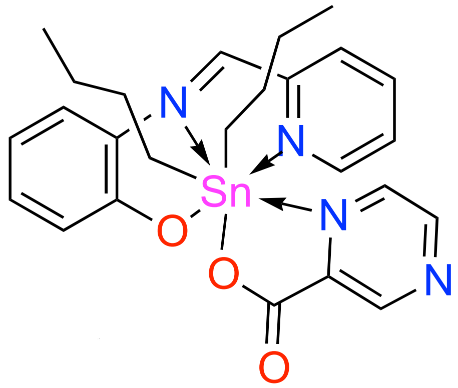

1a

Figure S7. HSQC spectrum of **1a**.

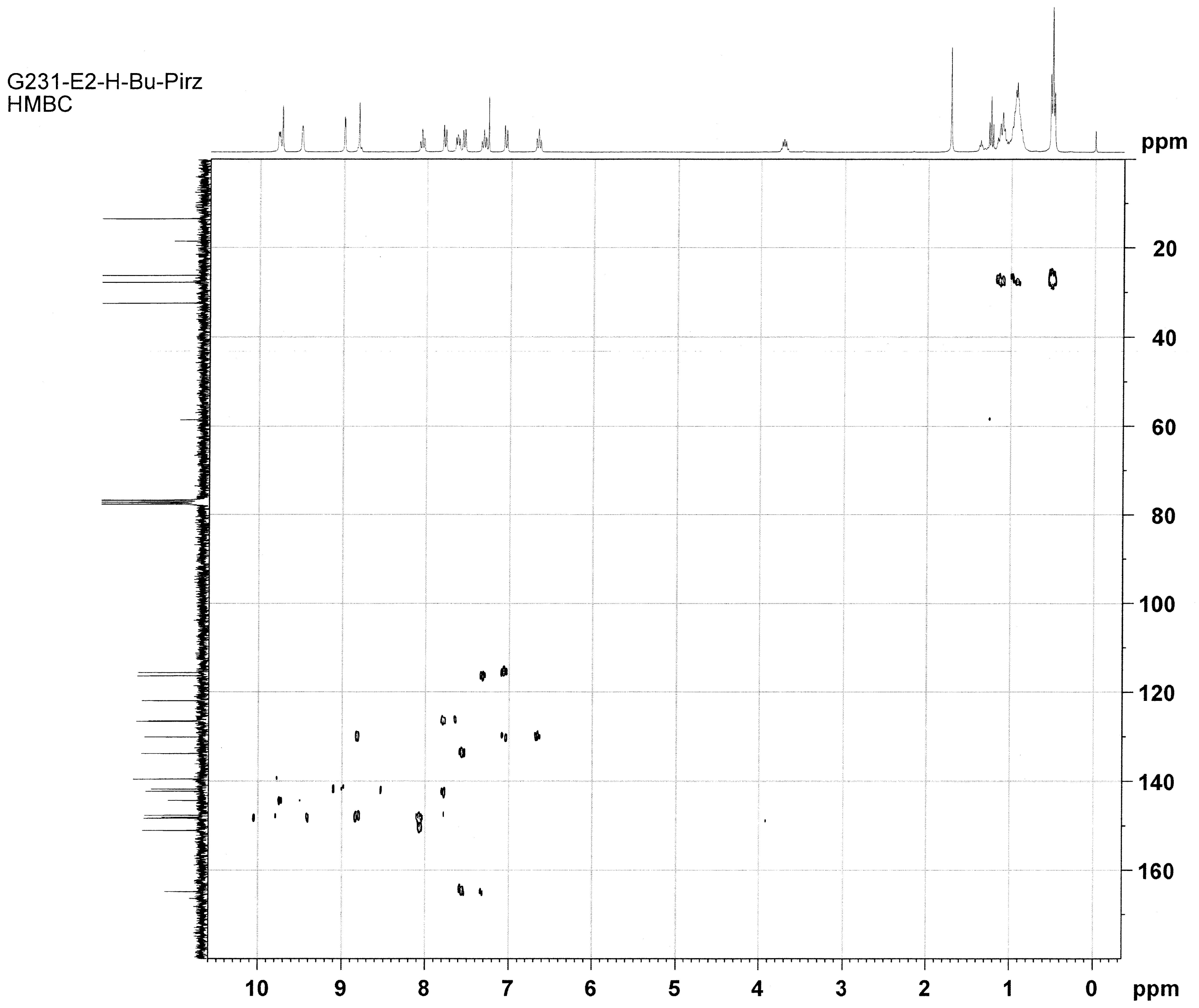

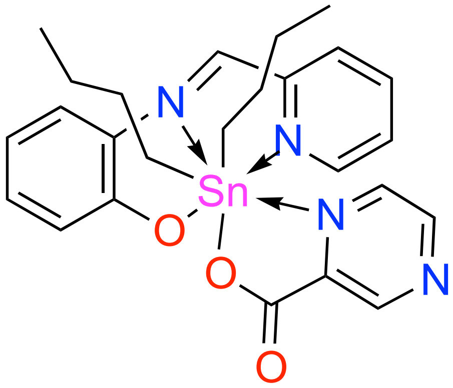

1a

Figure S8. HMBC spectrum for **1a**

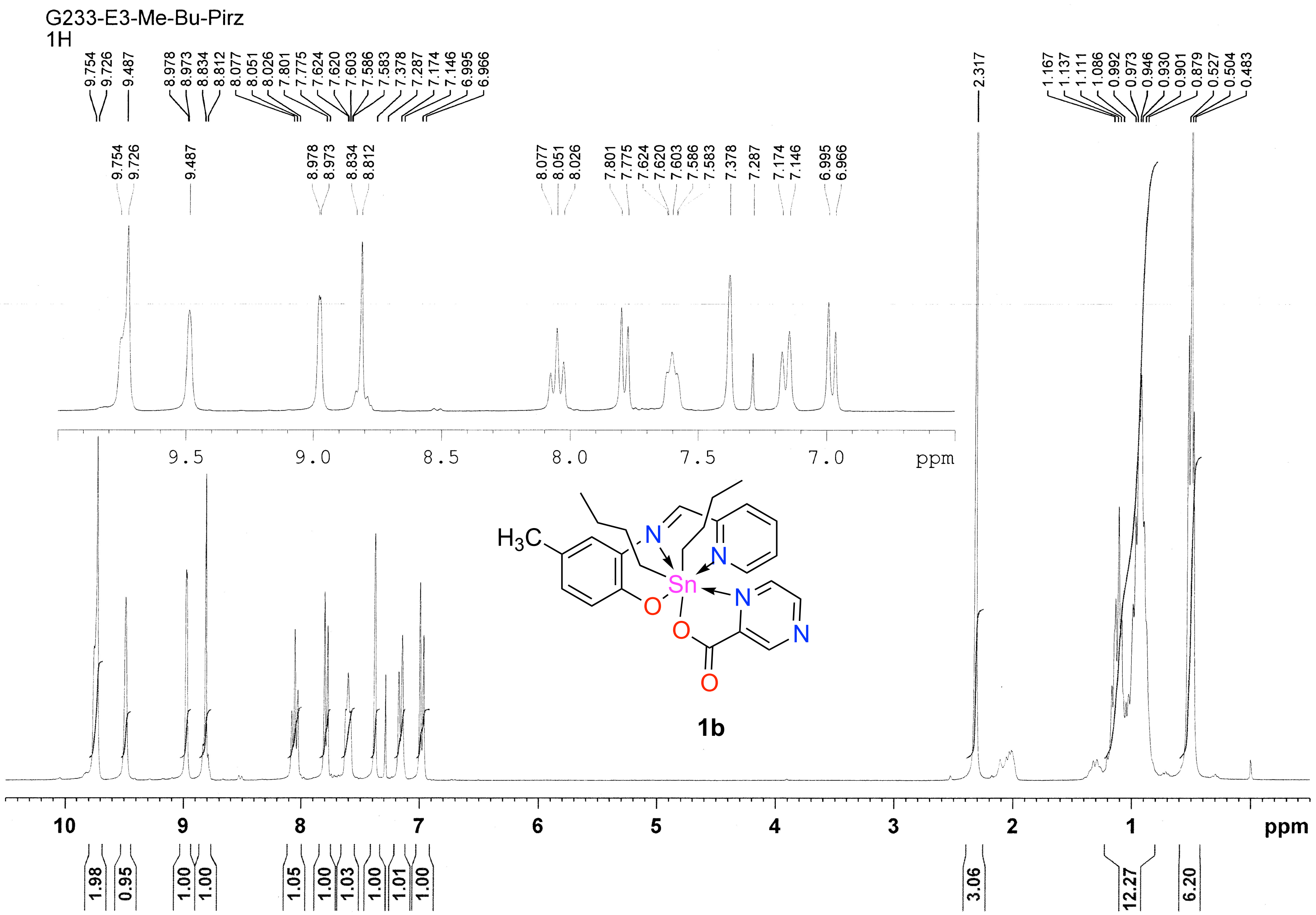

Figure S9. ^{1}H NMR spectrum of **1b**.

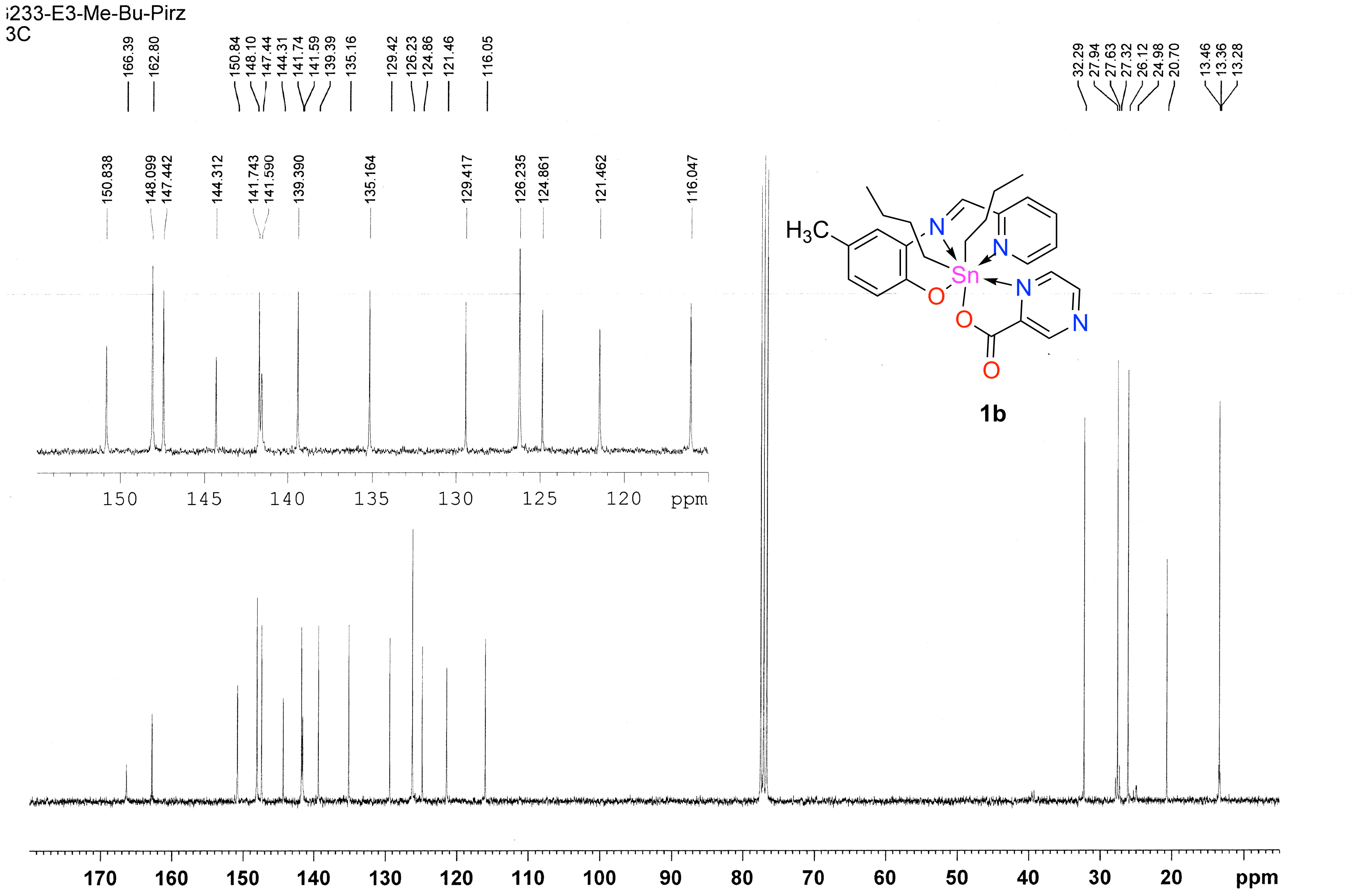

Figure 10. ^{13}C NMR spectrum of **1b**.

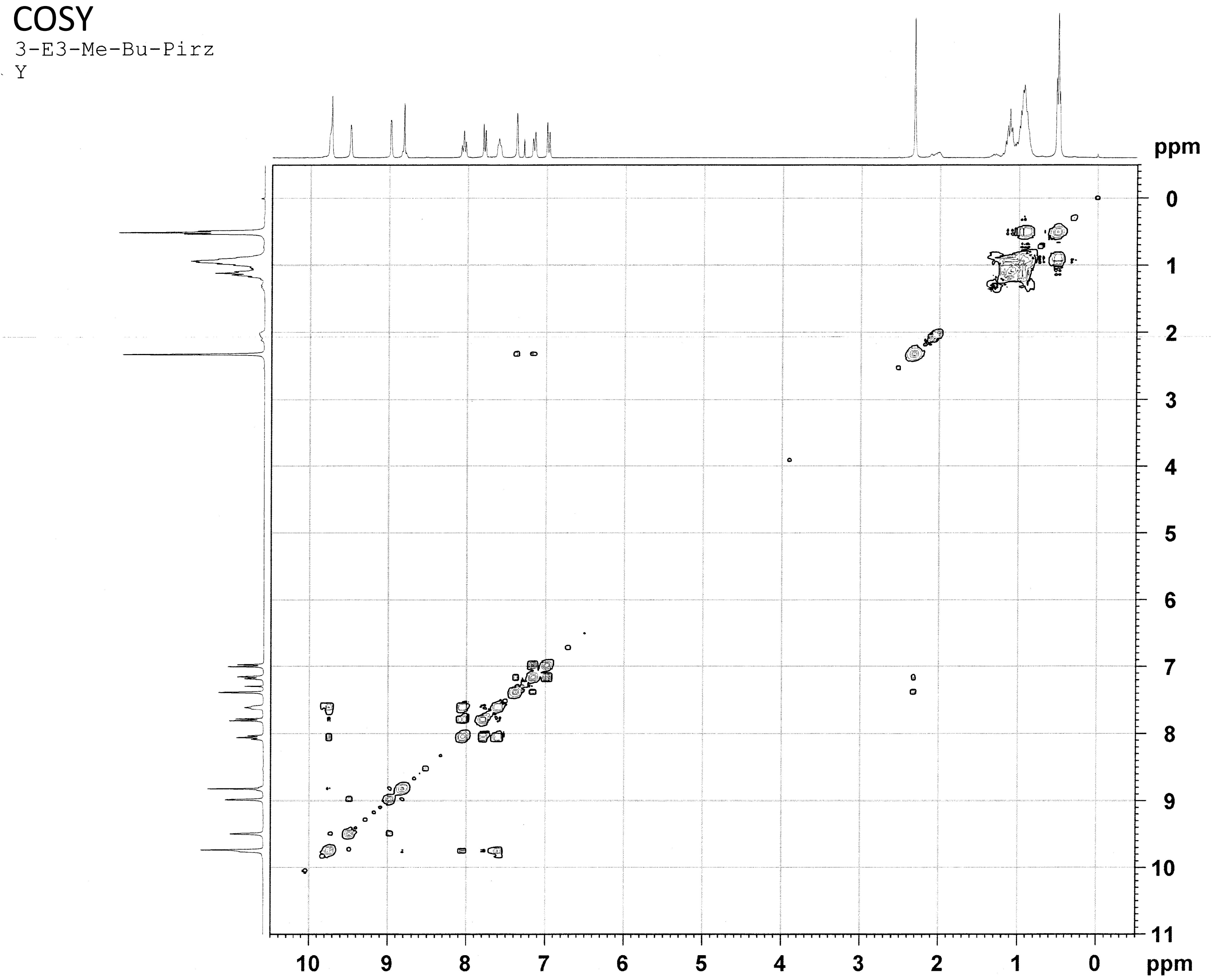

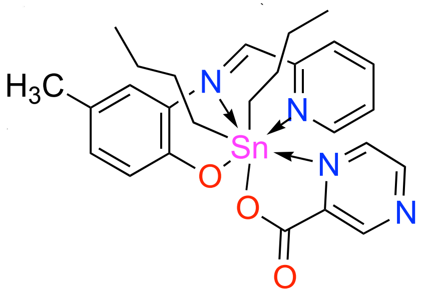

1b

Figure S11. COSY spectrum of **1b**.

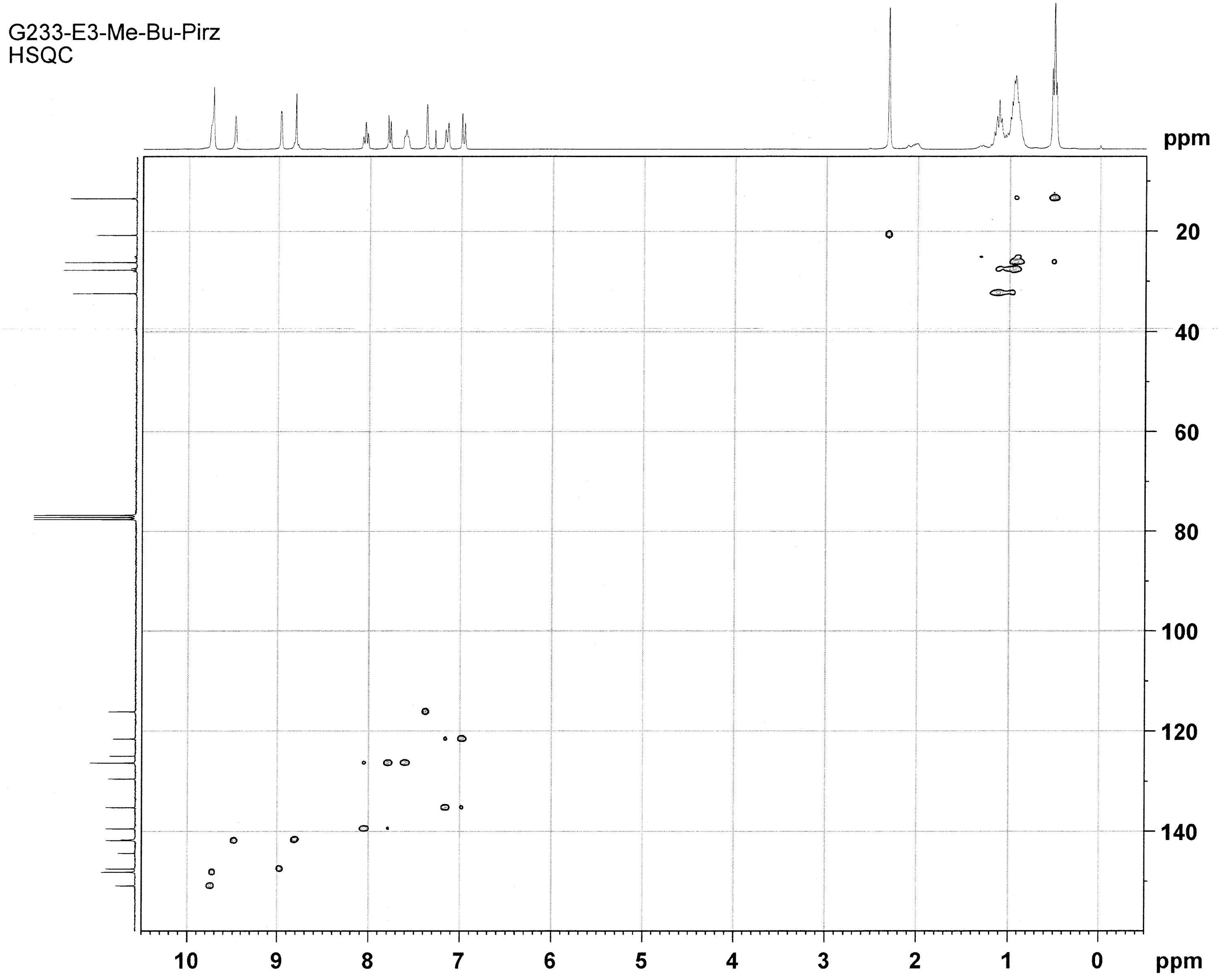

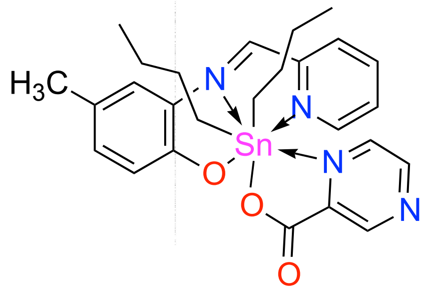

1b

Figure S12. HSQC spectrum of **1b**.

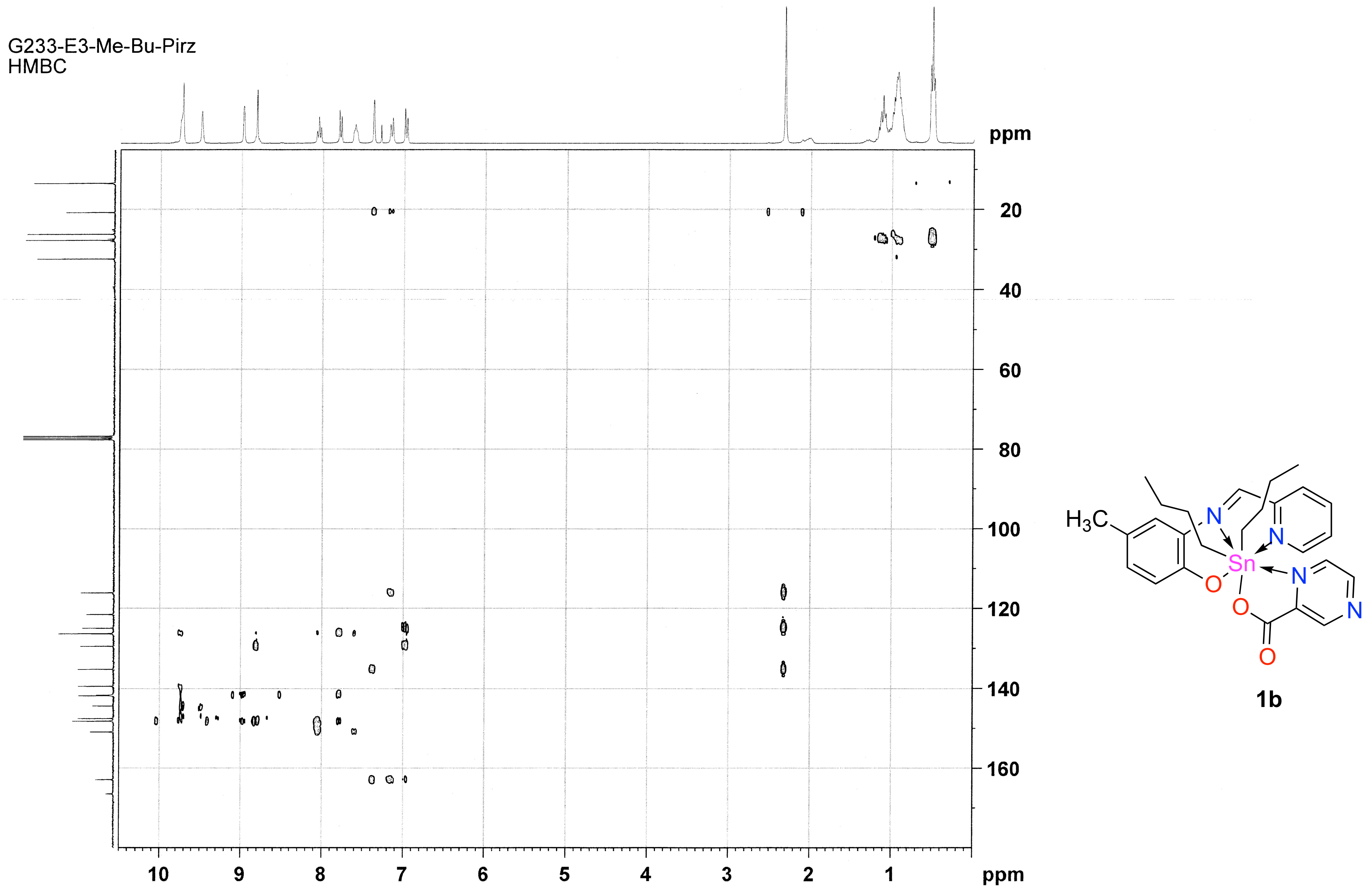

Figure S13. HMBC spectrum of **1b**.

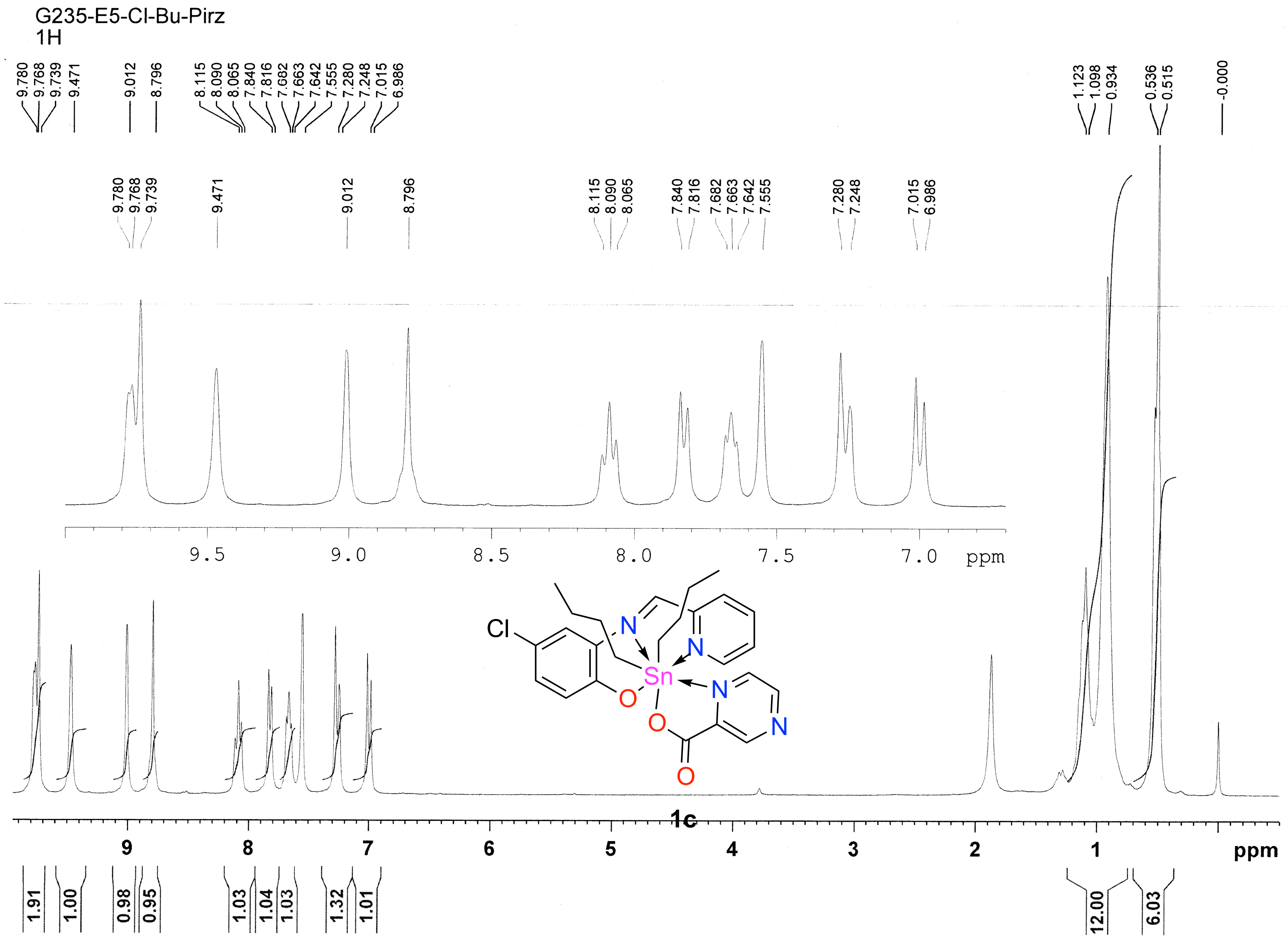

Figure S14. ^{1}H NMR spectrum of **1c**.

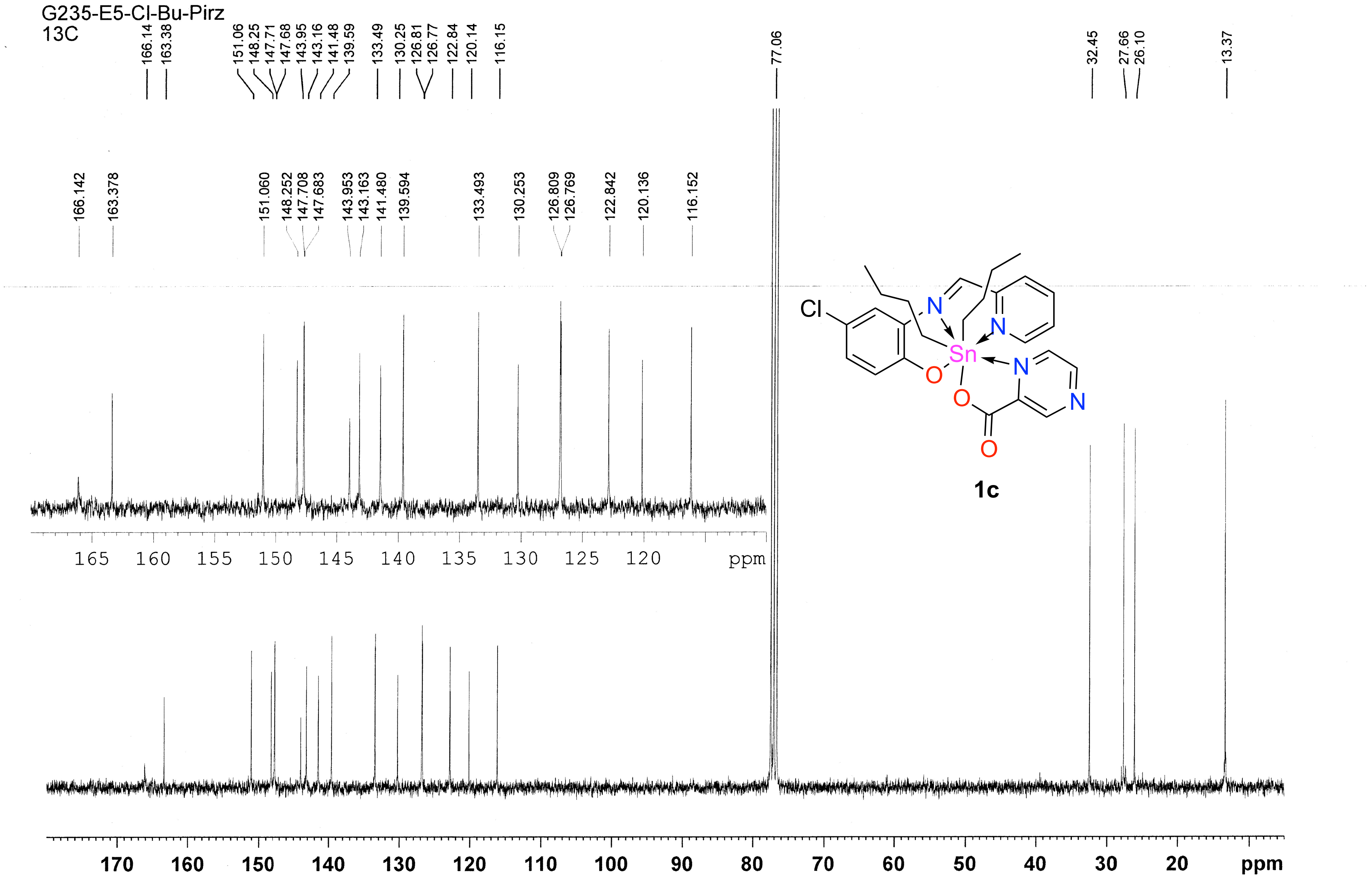

Figure S15. ^{13}C NMR spectrum of **1c**.

G235-E5-Cl-Bu-Pirz
COSY

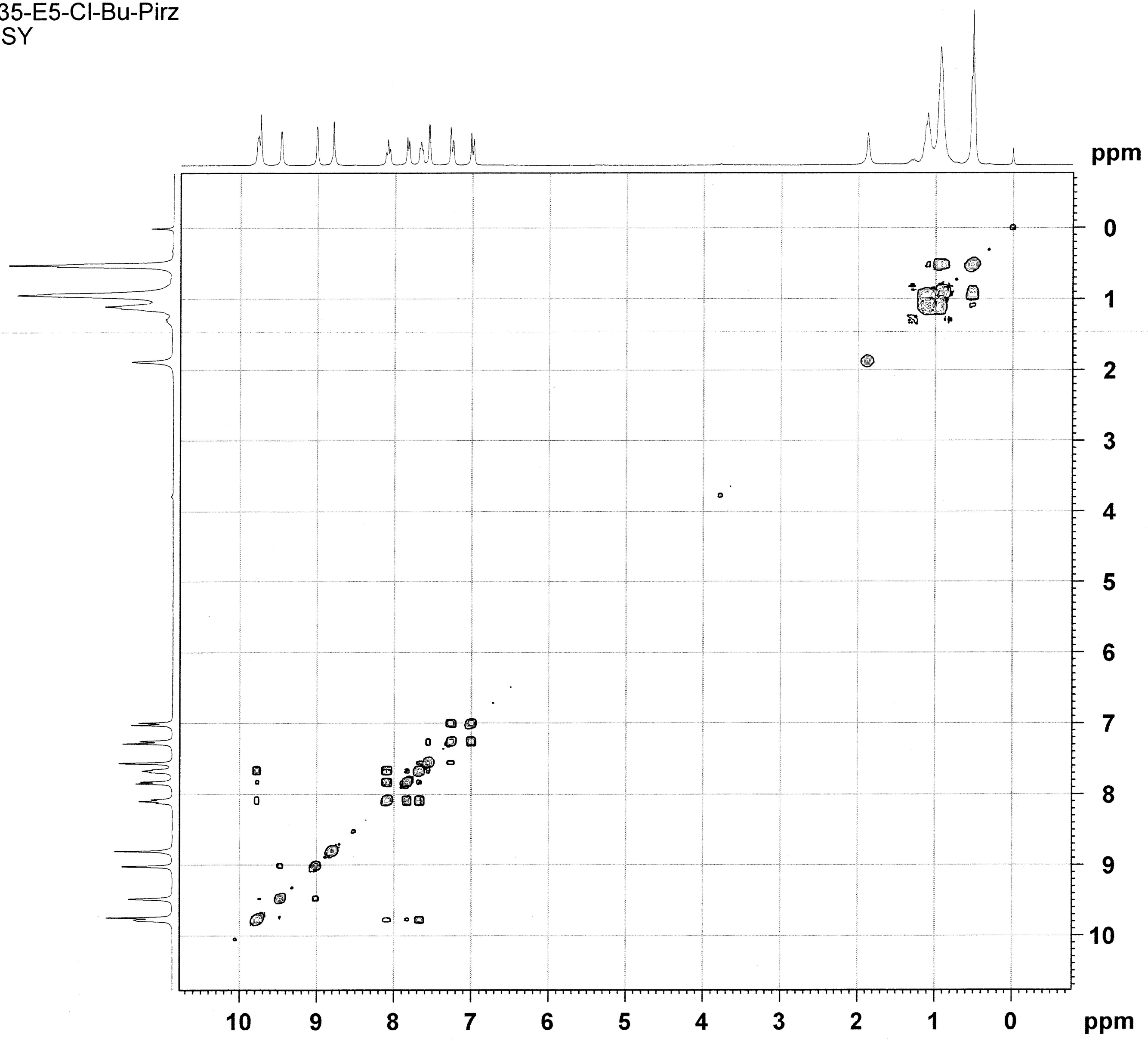

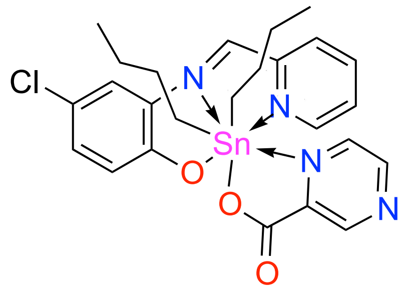

1c

Figure S16. COSY spectrum of **1c**.

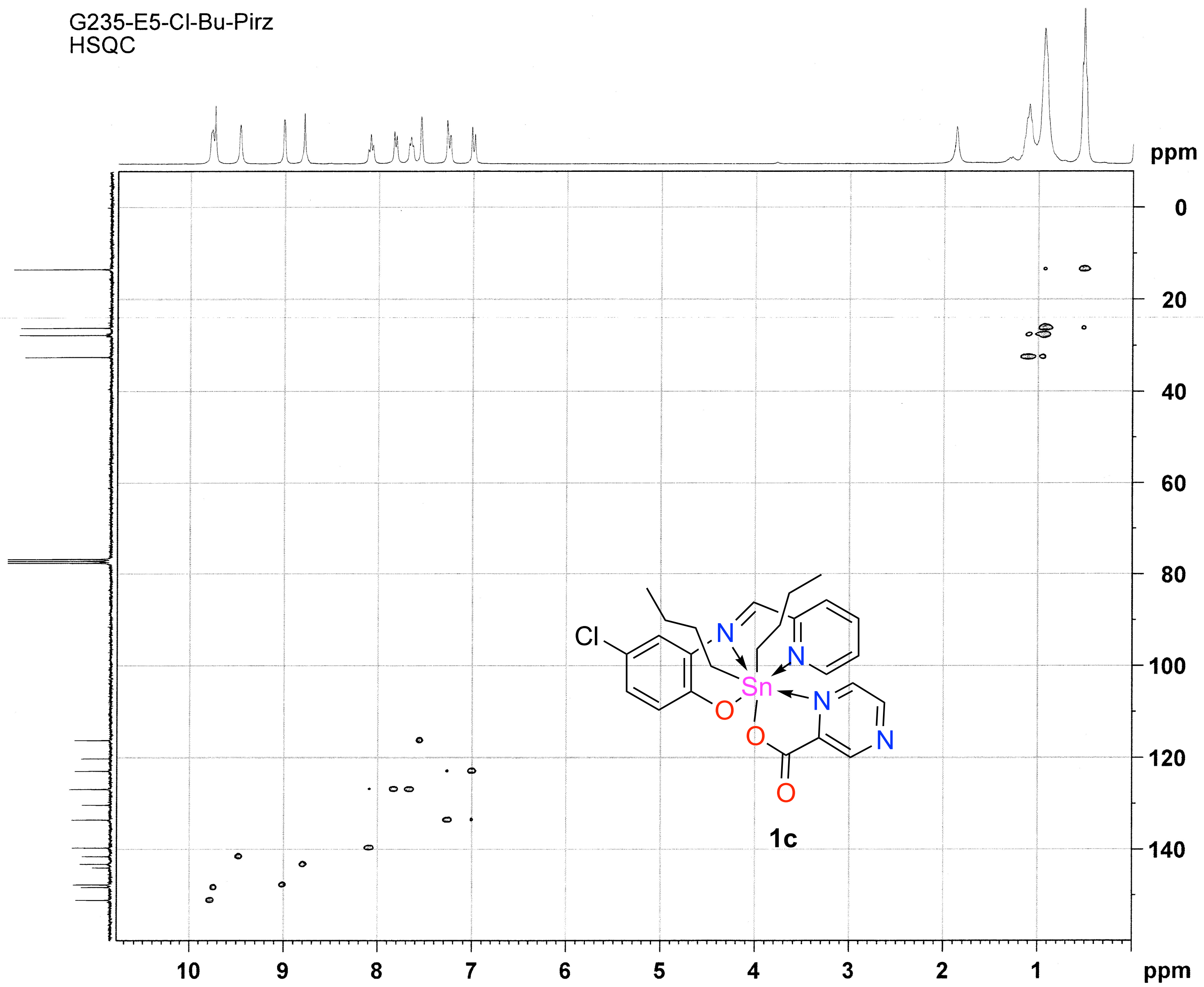

Figure S17. HSQC spectrum of **1c**.

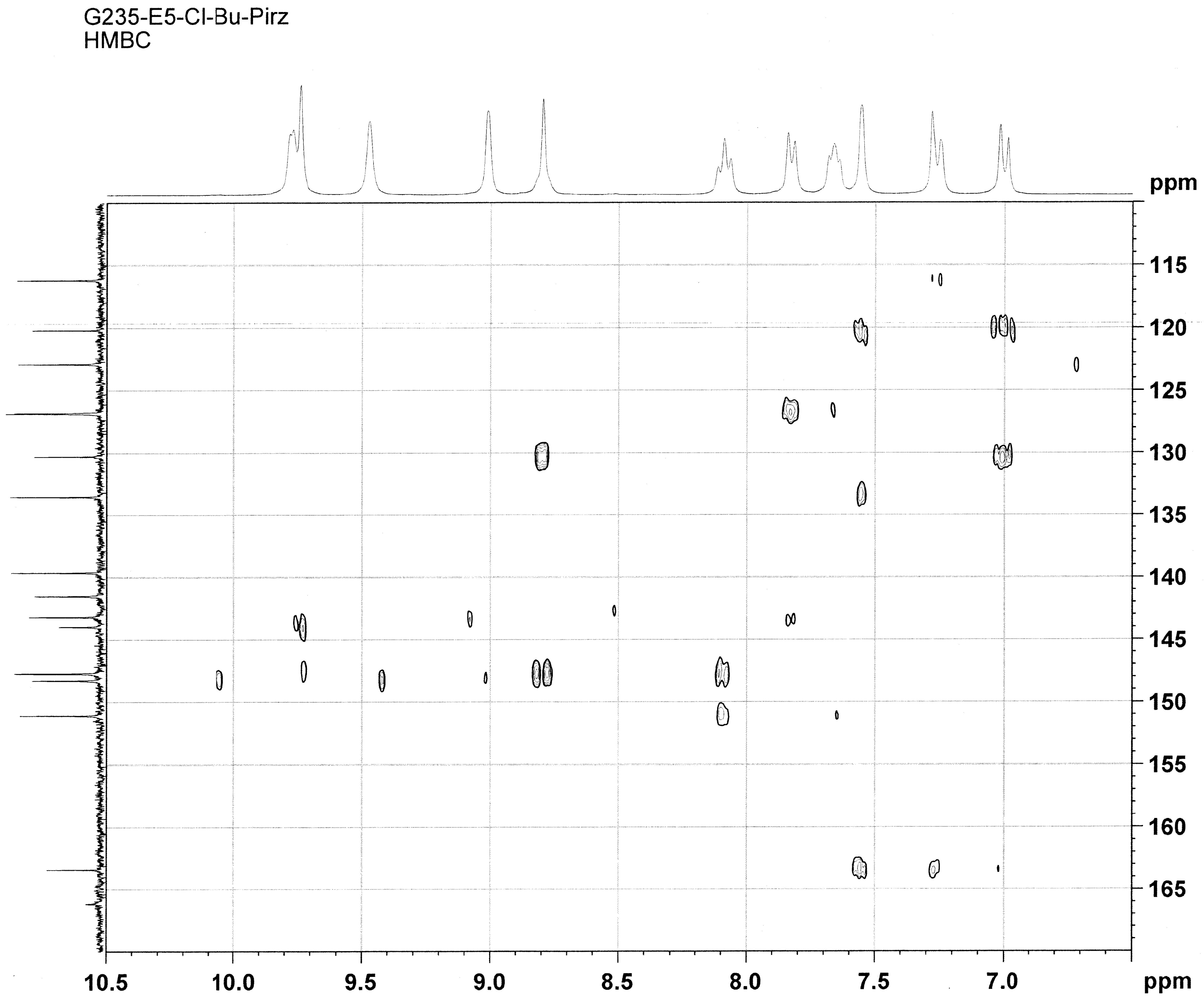

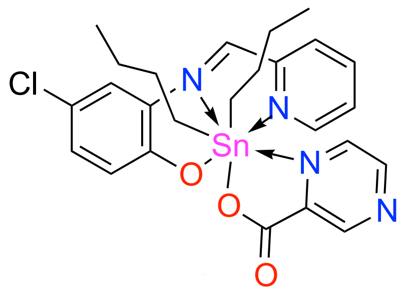

1c

Figure S18. HMBC spectrum of **1c**.

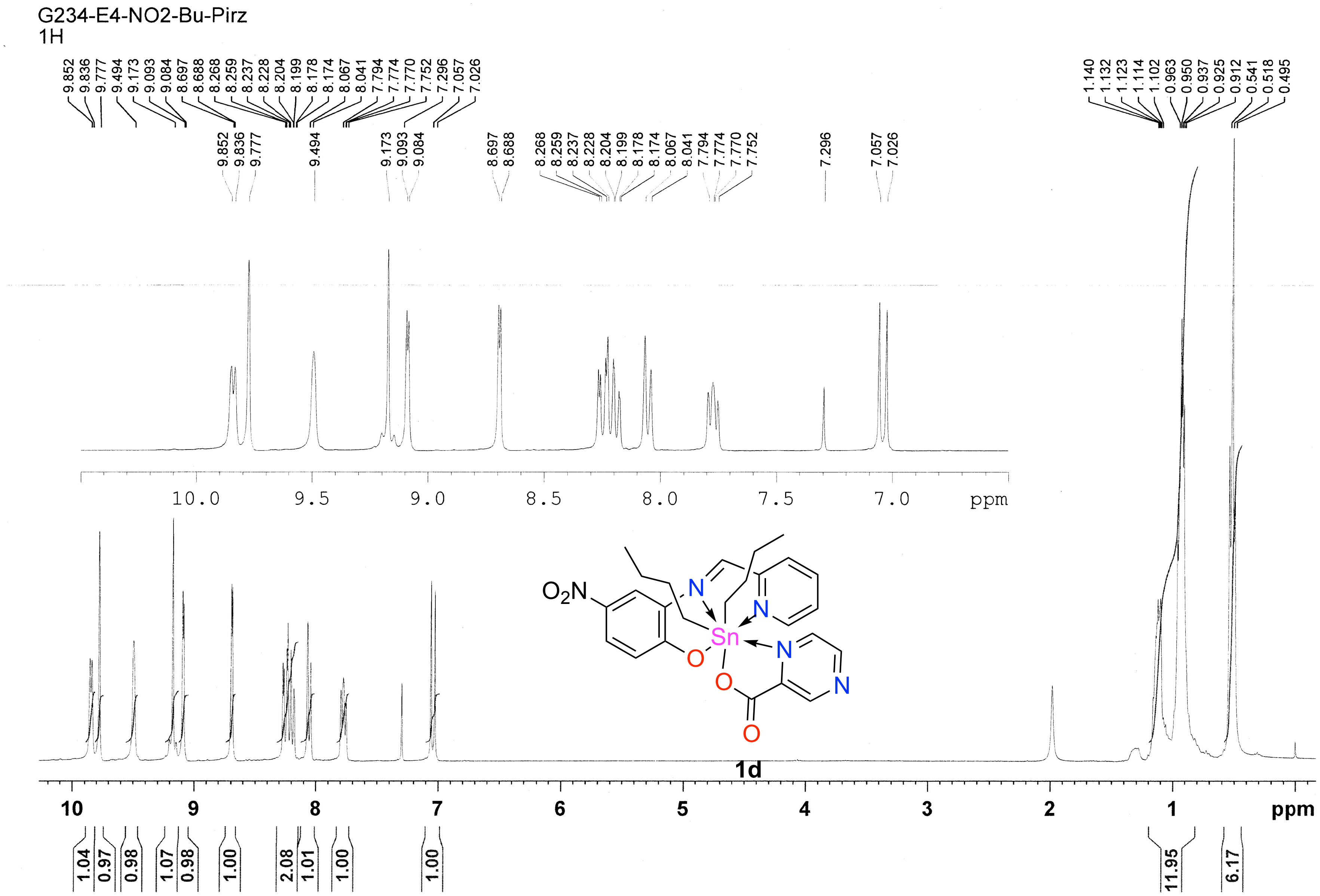

Figure S19. ^{1}H NMR spectrum of **1d.**

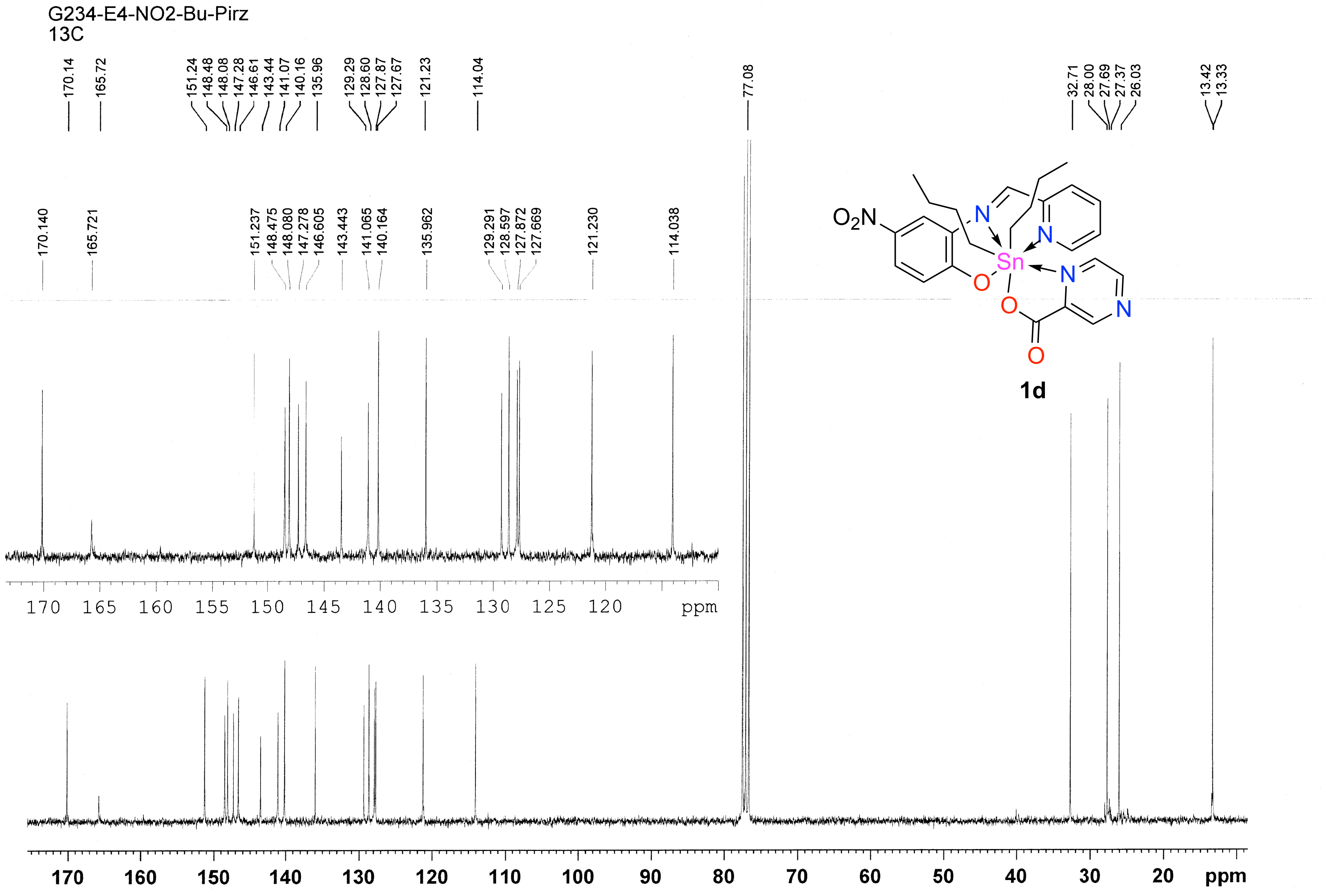

Figure S20. ^{13}C NMR spectrum of **1d**.

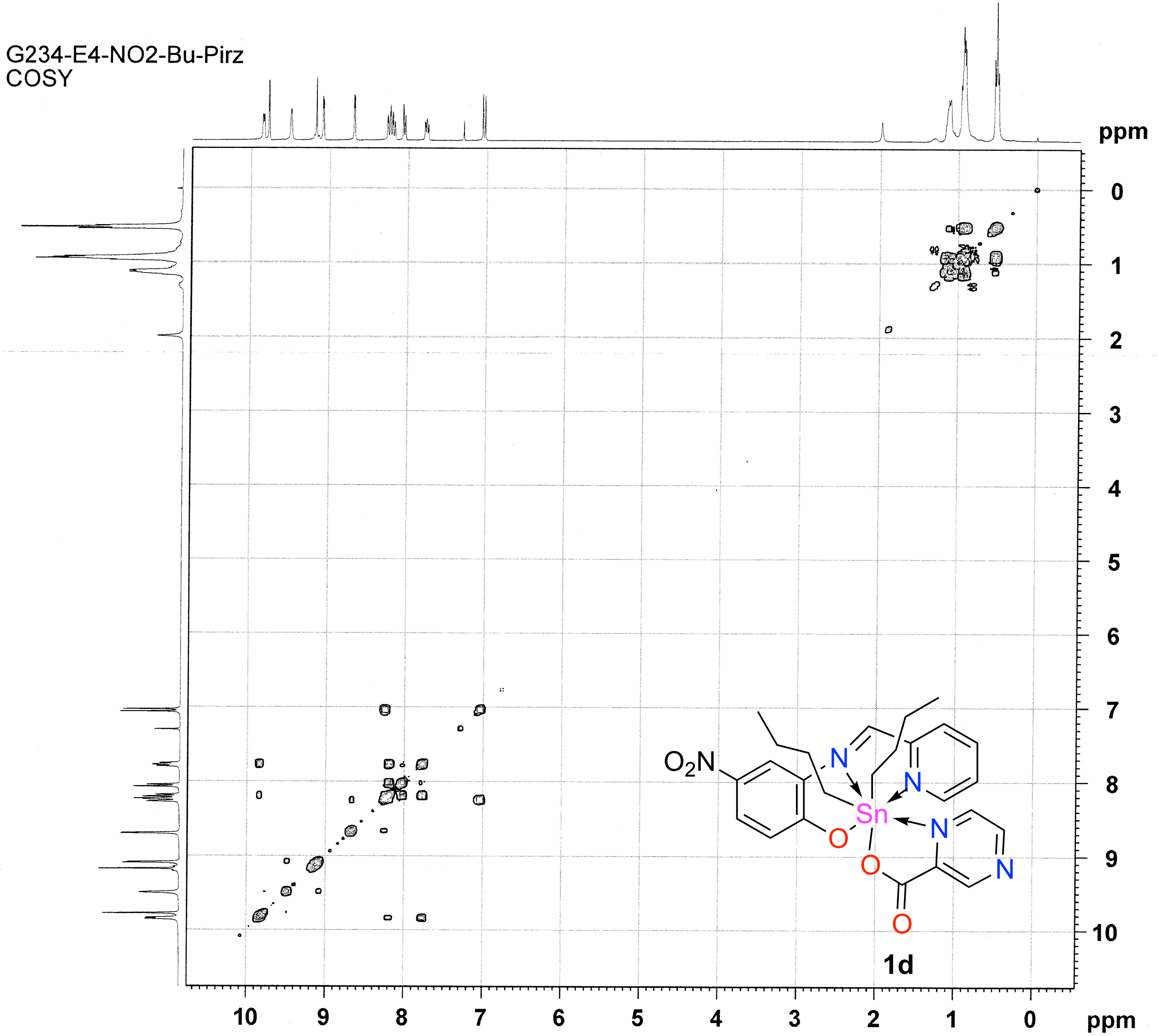

Figure S21. COSY spectrum of **1d**.

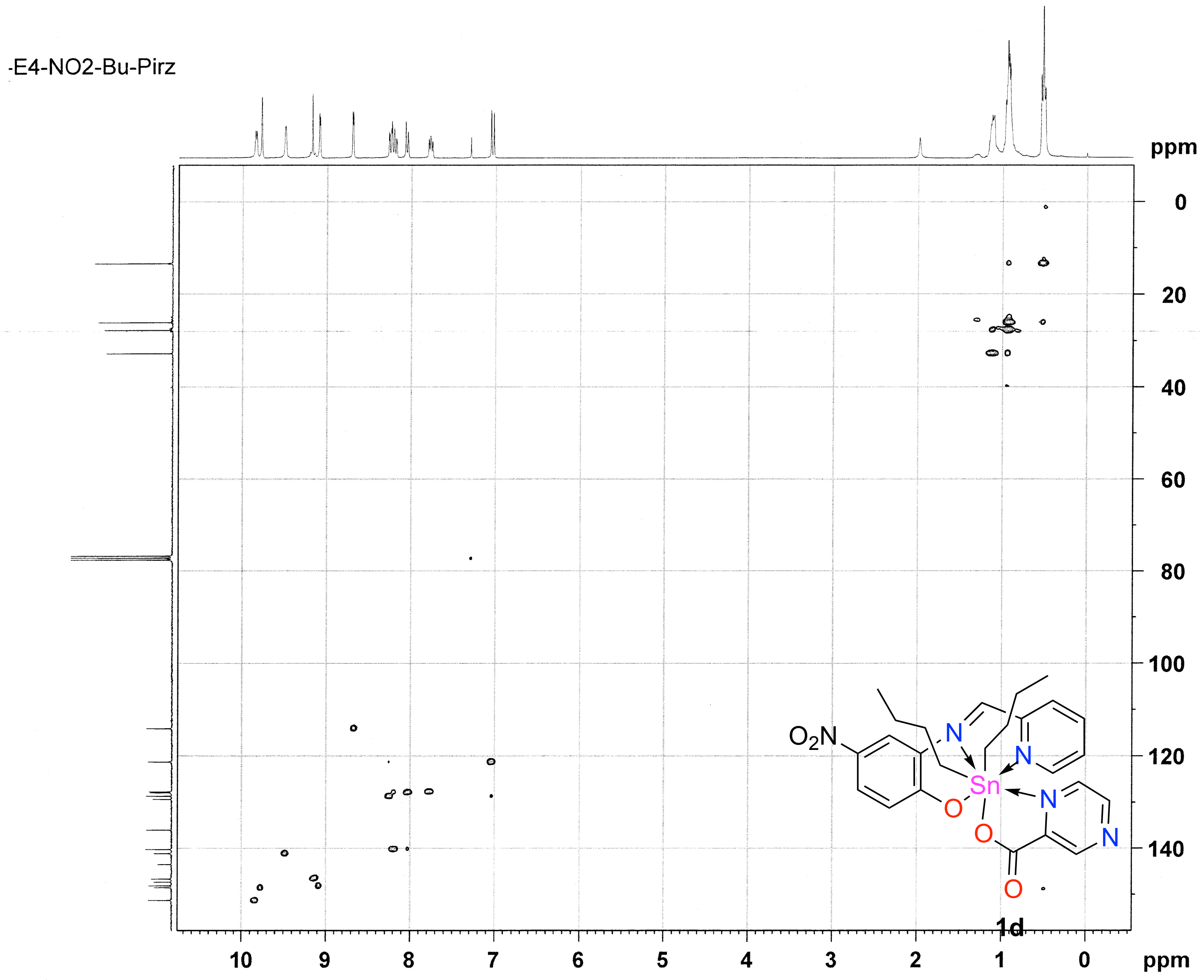

Figure S22. HMBC spectrum of **1d**.

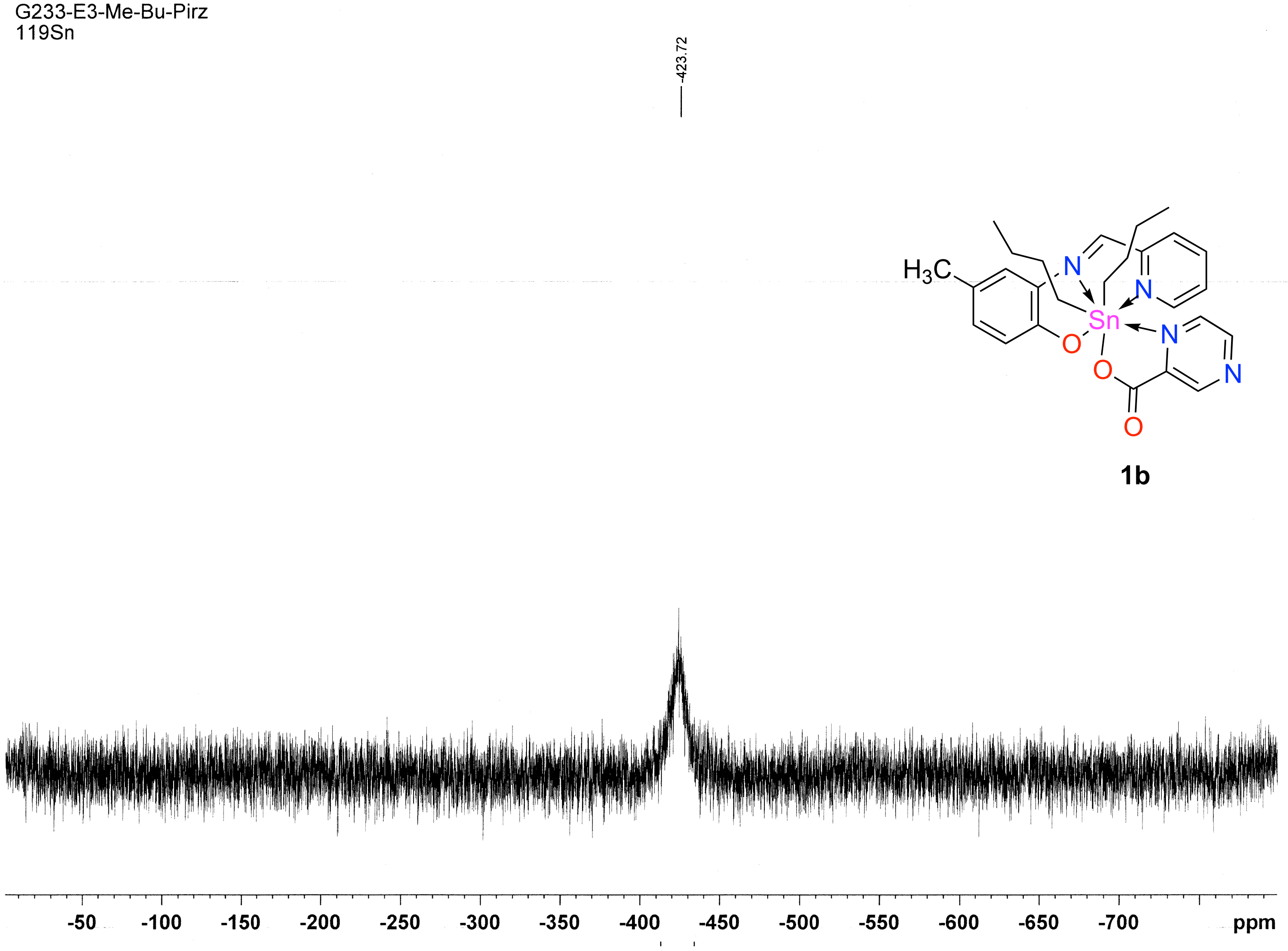

Figure S23. ^{119}Sn NMR in $CDCl_3$ solution of **1b**.
